# Exploring the developmental mechanisms of tea plant trichomes using genomics and single-cell transcriptome sequencing

**DOI:** 10.1093/hr/uhaf352

**Published:** 2025-12-09

**Authors:** Xuming Deng, Yajun Tang, Qing Zhang, Weilong Kong, Xiying Lin, Xianyu Chen, Zhidan Chen, Xingtan Zhang, Weijiang Sun

**Affiliations:** College of Horticulture, Fujian Agriculture and Forestry University, Fuzhou, Fujian 350002, China; Fruit Research Institute, Fujian Academy of Agricultural Sciences, Fuzhou, Fujian 350013, China; State Key Laboratory for Conservation and Utilization of Subtropical Agro-Bioresources, College of Agriculture, Guangxi Key Laboratory of Sugarcane Biology, Guangxi University, Nanning, China; National Key Laboratory for Tropical Crop Breeding, Shenzhen Branch, Guangdong Laboratory for Lingnan Modern Agriculture, Genome Analysis Laboratory of the Ministry of Agriculture, Agricultural Genomics Institute at Shenzhen, Chinese Academy of Agricultural Sciences, Shenzhen, Guangdong 518120, China; Tea Technology Promotion Station, Fuding City Tea Industry Development Center, Ningde, Fujian 355200, China; Tea Technology Promotion Station, Fuding City Tea Industry Development Center, Ningde, Fujian 355200, China; Anxi College of Tea Science, Fujian Agriculture and Forestry University, Quanzhou, Fujian 362400, China; National Key Laboratory for Tropical Crop Breeding, Shenzhen Branch, Guangdong Laboratory for Lingnan Modern Agriculture, Genome Analysis Laboratory of the Ministry of Agriculture, Agricultural Genomics Institute at Shenzhen, Chinese Academy of Agricultural Sciences, Shenzhen, Guangdong 518120, China; College of Horticulture, Fujian Agriculture and Forestry University, Fuzhou, Fujian 350002, China

## Abstract

*Camellia sinensis* Fuding Dahaocha, a triploid white tea cultivar widely cultivated in south China, exhibits distinctive traits including dense leaf trichomes, early sprouting, and robust stress resistance. Here, we present the first high-quality chromosome-level genome assembly of this triploid variety, resolved through integrated PacBio long-read sequencing and Hi-C scaffolding. The genome assembly spans 45 chromosomes with a scaffold N50 value of 182 Mbp. A total of 149 455 gene models were annotated and mapped to chromosomes, among which 30 568 were identified as protein-coding genes. The genome features high repetitiveness (65.9% transposable elements), heterozygosity, and three distinct haplotype sets with substantial allelic variation (17 601 triallelic genes), with the retained haplotype-specific genes potentially contributing to regulatory complexity through dosage effects. Genome completeness assessment revealed a BUSCO completeness of 99.0% (2303 out of 2326 conserved core genes identified), which included 40 single-copy (1.7%) and 2263 duplicated (97.3%) genes. Evolutionary analyses indicated conserved relationships among the three homologous chromosome sets. We also performed single-nucleus RNA sequencing on a sufficiently large pooled sample of leaf tissues to study trichome development, overcoming technical limitations posed by secondary metabolites and low protoplast isolation efficiency. This yielded a single-cell atlas for woody plants, identifying 35 trichome-specific marker genes and modeling developmental trajectories during epidermal differentiation. Functional validation identified CsCUT1 as a suppressor of trichome branching and CsMYB4 as a negative regulator of trichome initiation. Cell cycle analysis showed G2-phase dominance in developing trichomes. These findings provide a genetic framework for trichome development and offer resources for tea breeding.

## Introduction

Tea plant (*Camellia sinensis*), a globally significant beverage crop renowned for its unique aroma, flavor, and rich bioactive compounds, holds paramount importance in the food and pharmaceutical industries [1, 2]. With the rapid advancements in biotechnology, multidisciplinary studies related to tea plants have garnered widespread attention, particularly investigations into the tea plant genome, offering novel perspectives on its origin, evolution, genetic improvement, and mechanisms underlying quality traits [3]. Polyploidy, a common phenomenon in the plant kingdom, often confers vigorous growth, stress tolerance, and hybrid vigor advantages [4]. In tea plants, polyploidy similarly exerts profound effects on growth, development, and quality attributes [5]. Therefore, in-depth exploration of the tea plant polyploid genome holds significant implications for unraveling its genetic basis, expediting breeding programs, and enhancing tea quality. However, despite some progress in studying polyploidy in tea plants, there remains a dearth of reports on high-quality polyploid genomes in tea plants. This deficiency partly hinders our comprehensive understanding of polyploid inheritance in tea plants and impedes further advancements in tea breeding efforts. Thus, urgent initiatives are needed to undertake high-quality sequencing and analysis of the tea plant polyploid genome to address this research gap.

Trichomes on tea plant leaves, akin to those in *Arabidopsis thaliana*, exhibit a typical unicellular structure and belong to nonglandular trichomes [6]. As an essential part of tea leaves, tea trichomes are vital for the appearance, aroma, taste, and nutrition profile of tea. During processing, these substances are released and act as key precursors in the formation of tea aroma. Tea trichomes are rich in volatile aromatic compounds and a high level of amino acids, which are crucial for tea flavor, determine the freshness of the tea soup, and directly impact its taste quality. Notably, the dense trichome coverage characteristic of FDDH has been empirically associated with enhanced resistance to piercing-sucking pests such as tea green leafhoppers under field cultivation conditions. Trichomes originate from the development of epidermal pavement cells and generally undergo three stages: initiation induction, endoreduplication (a process in which the genome undergoes repeated rounds of replication without cell division, leading to elevated ploidy levels), and expansion with morphogenesis [7, 8]. While other cells in the epidermis continue mitotic division, precursor cells of trichomes cease the mitotic cycle upon determining cell fate, transitioning to the endoreduplication phase characterized by chromosome polyploidization [9, 10]. Theoretically, all epidermal cells possess the capability to initiate trichome differentiation during this stage. The regulatory network governing trichome initiation revolves around three proteins: GLABRA 3 (GL3), GLABRA 1 (GL1), and TRANSPARENT TESTA GLABRA 1 (TTG1), with both positive and negative regulatory feedback mechanisms present [11–13]. However, current research on trichomes in tea plants primarily focuses on deciphering the regulatory roles of individual genes or gene families, with the specific regulatory network of trichome development at the single-cell level remaining unclear. Furthermore, the formidable technical challenges associated with single-cell RNA sequencing in woody perennials, such as recalcitrant cell walls and abundant secondary metabolites, have thus far prevented the construction of a high-resolution cellular atlas for tea. This gap is particularly salient in polyploid cultivars, where potential allelic interactions and gene dosage effects could play decisive roles in trichome development, mechanisms that remain entirely inaccessible through conventional bulk sequencing approaches. Consequently, the regulatory network underlying trichome development at single-cell resolution in polyploid tea represents a critical and unexplored frontier for understanding the genetic basis of its specialized traits.

Cells constitute the fundamental structural and functional units of organisms, forming a hierarchically organized system through interdependence and interaction [14]. Traditional studies often analyze entire tissues and obtain average values of all cell information within the tissue, thereby masking the essence of cellular heterogeneity. Single-cell transcriptomic sequencing (scRNA-seq) has begun to emerge in plant research by exploring the developmental differentiation of different cell types and responses to abiotic stress. Wang *et al.* [1, 2] constructed the first single-cell transcriptome of tea plant leaf tissues and elucidated the specificity of genes involved in certain metabolite biosynthesis during cell development. However, due to limitations of protoplast extraction methods, in-depth single-cell sequencing of trichome tissues in tea leaves and the construction of a comprehensive single-cell atlas of leaves have not yet been achieved.

*Camellia sinensis* Fuding Dahaocha (FDDH) is a triploid asexually cultivated variety characterized by its small arbor-type tree structure, belonging to the large-leaf category and exhibiting early bud sprouting. This variety serves as a primary cultivar for white tea production, characterized by its densely distributed and abundant trichomes on the young leaves, robust plant structure, exceptional resistance, high yield characteristics, and widespread cultivation across various regions in southwestern China. In this study, we obtained a high-quality genome of the triploid *C. sinensis* FDDH using a combination of Hi-C and third-generation PacBio sequencing methods. To elucidate the single-cell transcriptomic atlas and regulatory dynamics of trichomes on tea leaves, we performed single-nucleus RNA sequencing (snRNA-seq) on tea leaf tissues and trichomes, exploring gene expression patterns. Our study presents the first dynamic map of epidermal cell differentiation into trichomes and guard cells, providing a unique perspective on the response mechanisms underlying trichome development at the single-cell level.

## Results

### Assembly and annotation of a triploid genome

**Figure 1:**
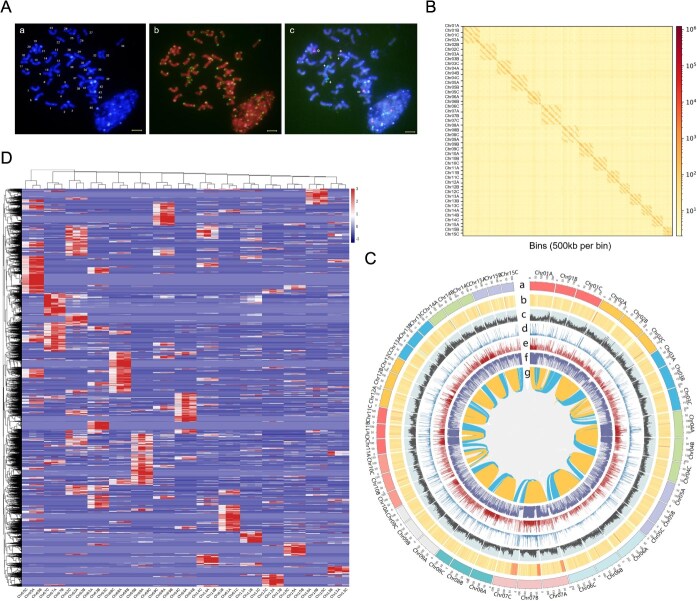
Overview of karyotype analysis and genome of *C. sinensis* FDDH. (A) Karyotype pictures of *C. sinensis* FDDH using (a) DAPI staining, (b) FISH with a telomere-specific repeat probe, and (c) 5SrDNA and 18SrDNA repeat sequence probe. 5SrDNA hybridization signals are marked with arrows, and 18SrDNA hybridization signals are marked with asterisks. Bar = 5 μm. (B) The Hi-C interaction heatmap plot of *C. sinensis* FDDH genome. Note: The interaction signal is indicated by the heatmap. The red color density is correlated with interaction intensity. The x-axis represents the position of the bin with a window size of 500 kb, and y-axis represents the homolog chromosome set from 1 to 10 in *C. sinensis* FDDH. The 15 interaction squares represent the 15 homologous chromosome sets of *C. sinensis* FDDH. (C) The circle map of the genomic features of *C. sinensis* FDDH. Note: The tracks indicate (from outermost to innermost) (a) 45 pseudochromosomes in Mbp, (b) GC content distribution, (c) gene density, (d) SNP density, (e) InDel density, (f) TE density, and (g) colinearity between chromosomes. (D) Distribution of counts of specific 13-mers in the chromosomes. Note: The red color density is correlated with interaction intensity.

As *C. sinensis* FDDH is a highly self-incompatible dicotyledonous plant with a complex genetic background, a genome survey is required before embarking on genome assembly. We conducted chromosome karyotyping analysis on its genome. More than 20 root cell chromosome samples were observed and recorded by applying the high-resolution metaphase chromosome preparation technique. By DAPI staining, we estimated that the genome of tea consisted of 45 chromosomes ranging from 4.0 to 6.0 μm in length (Fig. 1Aa) and was predominantly proximodistal mitotic chromosomes, many of which were enriched for telomeric heterochromatin. Chromosome numbers were confirmed by fluorescence *in situ* hybridization (FISH) and telomeric repeat probes, which showed a clear fluorescent signal at each telomere of the different chromosome ends (Fig. 1Ab). FISH analysis was then performed with 5SrDNA and 18SrDNA repeat sequence probes, in which three chromosomes had 5SrDNA signals (2 strong and 1 weak) and three chromosomes had 18SrDNA hybridization signals, finally confirming that the tea plant samples were triploid with a karyotype of 2*n* = 3*x* = 45 (Fig. 1Ac). In order to enhance the precision of evaluating the genome size of *C. sinensis* FDDH and to furnish a reference for subsequent assembly, flow cytometry technology was applied in this investigation. *Zea mays* L. var B73, possessing a genome size of 2.3 Gbp, served as the internal reference genome. Through the integration of triploid karyotype analysis results, the size of its monoploid genome was estimated to be approximately 2.72 ± 0.05 Gbp (Fig. S1), demonstrating close concordance with the previously predicted values.

In this study, tender leaves of *C. sinensis* FDDH were collected for PacBio third-generation library construction, yielding 177 Gbp of filtered CCS reads from six cells, providing an 18× coverage (Table S2). The corrected CCS reads exhibited an average length of 17.3 kbp. Using Canu (v1.9), *de novo* assembly of these corrected CCS reads produced 59 462 high-quality contigs, totaling 8.3 Gbp across three sets of chromosomes, closely matching the genome size estimated by flow cytometry. The N50 and N90 values were 1.13 Mbp and 39 kbp, respectively, with an average contig length of 1.7 Mbp and a maximum contig length of 9.6 Mbp, indicating high assembly quality (Table S3).

Subsequently, three Hi-C libraries were constructed using tender leaves of the tea plant, and paired-end 150-bp sequencing on the HiSeqTM X Ten platform generated 150 Gb of data, equivalent to approximately 45× coverage (Table S2). After aligning the high-quality Hi-C reads to the contig-level genome, a total of 3 023 917 024 paired-end reads were specifically mapped to the genome (Table S4), which were further utilized to assist genome assembly. Subsequently, we anchored the contigs of the genome using the ALLHiC, and manually corrected problematic regions. Ultimately, 45 high-quality chromosomes and a few unanchored contigs were obtained, grouped into 15 sets, each containing three chromosomes, with strong Hi-C interaction signals observed between chromosomes within each set (Fig. 1B), consistent with karyotype analysis results. The intensity of Hi-C interaction signals was strongest between adjacent positions on each chromosome, gradually weakening with increasing physical distance (Fig. S2), reflecting the effectiveness of Hi-C-assisted assembly for the genome. Evaluation of the genome assembled using Hi-C revealed detailed statistics, with scaffold N50 and N90 values of 182 and 0.4 Mbp, respectively. The largest scaffold was 288 Mbp, and the average scaffold length was 0.4 Mbp. A total of 59 462 contigs were anchored to the chromosomes of *C. sinensis* FDDH, with 149 455 gene models present on the 45 chromosomes, accounting for approximately 80.36% of the total, with gene numbers ranging from 1961 to 4977 on each chromosome (Table S5). And we identified 30 568 genes, with 17 601 genes containing three alleles and 9239 genes containing two alleles (Table S3). Additionally, to systematically characterize the genomic features of *C. sinensis* FDDH, we generated a genome chromosome map (Fig. 1C) and conducted comparative statistical analyses of each chromosome’s characteristics. The three sets of homologous chromosomes exhibit certain differences, with the number of SNPs between chromosomes ranging from 7256 to 56 509, and the variation of InDels (0–10 bp) ranging from 1968 to 9371 (Table S6). Meanwhile, the content of transposable elements (TEs) within each chromosome ranges from 89.36 to 244.87 Mbp, accounting for as much as 65.9%. These results indicate that the genome of *C. sinensis* FDDH is highly repetitive and possesses a complex genome with a certain degree of heterozygosity.

We assessed the assembly quality of the *C. sinensis* FDDH genome using CEGMA and BUSCO software. BUSCO evaluates genome completeness by quantifying the percentage of conserved single-copy orthologs present in the assembly, providing a standardized metric for assembly quality assessment. The evaluation by CEGMA revealed that out of 248 eukaryotic core genes, 204 genes were identified in the genome, with 142 genes being completely annotated, accounting for 88.03% and 57.26% of the core gene set, respectively, resulting in a genome completeness of 95.10% (Table S7). However, due to the conservative nature of CEGMA’s core gene set and their relatively short length, as well as their poor specificity, fewer genes were completely annotated. Consequently, we further assessed the genome using BUSCO, which indicated that out of 2326 conserved core genes, 2303 genes were found in the *C. sinensis* FDDH genome, representing approximately 99.0%. Among them, 40 genes were complete single-copy BUSCO core genes, and 2263 genes were complete duplicated BUSCO core genes, accounting for 1.7% and 97.3%, respectively (Table S8). Notably, by examining the clustering patterns of 13-mers across the 45 chromosomes of *C. sinensis* FDDH, as shown in Fig. 1D, it is evident that the distribution of 13-mers on each chromosome is relatively uniform. Additionally, we collected population SNP data for the *Camellia* genus (196 cultivars) from Zhang *et al.* [15] and reconstructed a maximum likelihood tree that includes the three chromosomal groups of *C. sinensis* FDDH (Fig. S3). The analysis reveals that the genetic evolutionary distances between the chromosomal groups are highly similar. Based on these two lines of evidence, we conclude that *C. sinensis* FDDH is most likely a homologous triploid.

Collectively, this study reports the *de novo* assembly of a high-quality reference genome for the homologous triploid tea plant *C. sinensis* FDDH (2*n* = 3*x* = 45). Combining PacBio long-read sequencing (177 Gb data, ~18× coverage) and Hi-C technology, we generated a chromosome-scale assembly of ~8.3 Gbp, which is consistent with the flow cytometry-estimated monoploid genome size of ~2.72 Gbp. The assembly exhibits high continuity, with contig N50 and scaffold N50 values of 1.13 and 182 Mbp, respectively. A total of 59 462 contigs were anchored and oriented into 45 chromosomes, harboring 30 568 annotated genes with 149 455 gene models. Genome assessment revealed high completeness (95.10% by CEGMA; 99.0% by BUSCO) and uniform k-mer distribution. The genome is characterized by high repetitiveness (65.9% TEs) and heterozygosity, featuring three distinct haplotype sets with significant allelic variation (17 601 genes with three alleles). These haplotype-specific genes, which may represent either newly derived genes or evolutionarily conserved genes retained through natural selection, are proposed to enrich the regulatory network of tea plants via mechanisms such as dosage effects. The completion of this high-quality, chromosome-level genome assembly provided an essential reference for the subsequent scRNA-seq analysis, enabling precise alignment of sequencing reads and accurate quantification of allele-specific expression across different cell types.

### *‘Camellia sinensis* FDDH’ single-cell transcriptomic analysis

**Figure 2:**
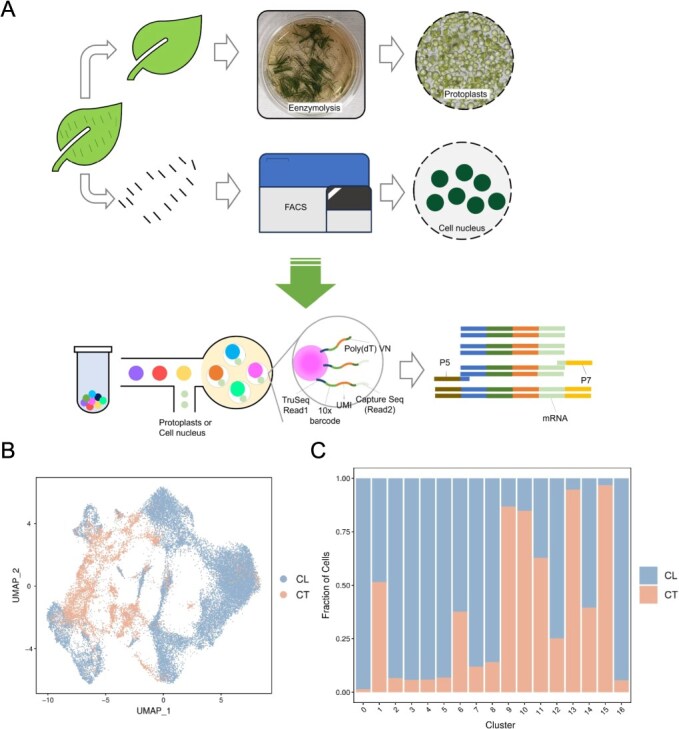
Single-cell transcriptome isolation and preliminary analysis of tea tree (*C. sinensis*) leaves. (A) 10× Genomics single-cell transcriptome sequencing process for *C. sinensis* FDDH. (B) Single-cell clustering UMAP plot of CL and CT groups. (C) Stacked map of cluster cells in each group.

The presence of abundant trichomes represents a staple trait in tea plants, particularly in *C. sinensis* FDDH. The density of trichomes on the tender shoots of tea bushes significantly influences the appearance and flavor profile of white tea. Existing methods for extracting protoplasts from tea leaf tissues have been reported as ineffective in isolating trichome cells. Hence, to delineate a comprehensive single-cell transcriptome atlas of *C. sinensis* FDDH leaves, we employed a dual approach involving native protoplast isolation and cell nucleus extraction for 10× Genomics scRNA-seq (Fig. 2A). This strategy not only maximizes the retention of transcriptional information from leaf cells but also captures the transcriptome details of trichome cell nuclei. In this study, we examined the scRNA-seq data from leaf and trichome samples of *C. sinensis* FDDH. Following the exclusion of low-quality sequencing reads, we conducted a thorough analysis of the read count and sequencing quality for each sample (Table S9). The number of high-quality reads identified across the three samples ranged from 0.211 to 0.433 billion, with over 95% representing valid barcodes.

Through the utilization of the Cell Ranger software, we meticulously aligned sequencing reads with the reference genome (*C. sinensis* FDDH genome) to establish associations between these reads and specific genes. Following this, we meticulously corrected and tallied Unique Molecular Identifiers (UMIs), culminating in an unfiltered feature-barcode matrix. Furthermore, using this matrix, cells and noncells in the dataset were accurately identified and differentiated, visually presented in barcode rank-plot plots (Fig. S4A) for stability and precision in effective cell identification. In cells of leaf-1 (CL-1), CL-2, and cells of trichome (CT), we discerned 9740, 16 974, and 7119 cells, respectively, each exhibiting median cell gene counts of 1147, 1065, and 2153. The fraction reads in cells for the three samples exhibited a range from 54.2% to 86.50%, while the high-confidence genome alignment rates ranged from 63.0% to 71.6%, underscoring the elevated data utilization and precision in alignment results (Table S10). Finally, predicated on the corrected UMIs and identified effective cells, we meticulously quantified gene expression within cells using UMI counts. Before embarking on the classification of cell clusters, we implemented a stringent filtering process to exclude anomalous cells, utilizing DoubletFinder for doublet removal. Additionally, cells were filtered based on specific criteria: the number of identified genes in a single cell should fall between 310 and 6000 (Fig. S4B), and the total UMI count in a single cell should not surpass 0.0 (Fig. S4C). Following this rigorous filtration, the cell counts in CT, CL-1, and CL-2 were 92.12%, 92.49%, and 87.02%, respectively, of the original count (Table S11).

### Generation of a comprehensive cell atlas of tea plant leaves

After applying Harmony batch effect correction to the merged samples of the three groups, we proceeded with soft k-means clustering on the dimensionality-reduced data. In this process, cells were probabilistically assigned to various clusters to maximize the diversity within each cluster’s dataset. Subsequently, we computed the global centroid for all datasets within each cluster, as well as the centroid for each specific dataset. Within each cluster, correction factors were calculated based on these centroids to adjust the cells, bringing them closer to the centroid. This iterative process continued until the clustering effect stabilized. Eventually, we obtained 17 single-cell clusters. Further, we employed the Uniform Manifold Approximation and Projection (UMAP) nonlinear clustering method to visualize the classification results of single-cell clusters. The number of cells in each cluster ranged from 127 to 7871 (Table S12), with CT group predominantly concentrated in clusters 1, 6, 9, 10, 11, 13, 14, and 15, while CL groups mainly clustered in other clusters (Fig. 2B and C).

**Figure 3:**
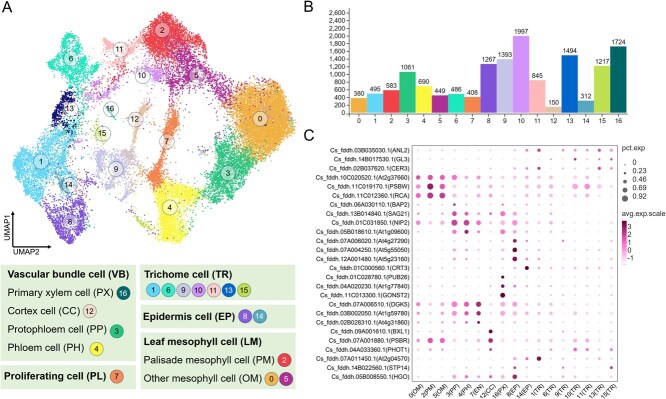
Cell heterogeneity in *C. sinensis* leaves. (A) UMAP plot of annotation results of cell clusters. (B) Statistical histogram of the number of significantly up-regulated genes in each cell cluster. (C) Bubble plot of marker gene expression distribution for identifying different cell types. Note: A larger circle in the legend indicates a larger proportion of the number of cells in that cluster expressing the gene and vice versa. Legend values represent the expression abundance of a gene, with larger values indicating a higher abundance of gene expression in that cell and vice versa.

Distinguishing cell types in nonmodel plants remains challenging. Understanding the molecular expression characteristics of various cell clusters is crucial. To achieve this, we applied the Seurat rank-sum test for analyzing differential gene expression in diverse cell clusters, quantifying the number of upregulated genes in each cluster. Among the 17 clusters (Fig. 3A), a total of 14 951 upregulated genes (highly variable genes, HVGs) were identified, providing a crucial basis for subsequent classification and annotation of each cluster (Fig. 3B). Subsequently, the selected upregulated genes were compared with marker genes for different cell types in the PlantCellMarker database (primarily based on *Arabidopsis*). This comparison facilitated the recognition and correction of cell types for each cell cluster, resulting in the identification of 26 important marker genes with homologous sequences to *Arabidopsis* in *C. sinensis* FDDH (Fig. 3C and Table S13). In this experiment, four major cell types were identified: vascular bundle cells (VB), leaf mesophyll cells (LM), epidermis cells (EP), and trichome cells (TR). VB included primary xylem cells (PX) from cluster 16, cortex cells (CC) from cluster 12, endodermis cells (EN) from cluster 7, protophloem cells (PP) from cluster 3, and phloem cells (PH) from cluster 4. LM comprised the cluster 2, palisade mesophyll cells (PM), and other mesophyll cells (OM) formed by the cluster 0 and 5. EP encompassed clusters 8 and 14. Combining the biological significance of experimental samples and the results of marker gene comparison, TRs were mainly composed of clusters 1, 6, 9, 10, 11, 13, and 15 (Fig. 3A).

**Figure 4:**
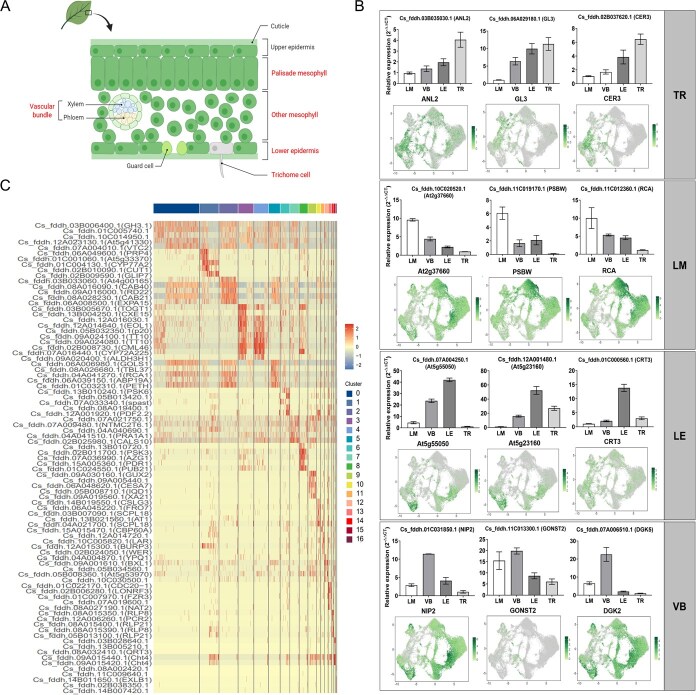
Identification of genes involved in *C. sinensis* leaves and new marker genes. (A) Schematic diagram of the cross-section of *C. sinensis* leaves. (B) RT-PCR quantification results and UMAP plots of marker genes in four leaf tissues. Note: TR indicates trichome cell; LM, leaf mesophyll; LE, lower epidermis of leaf; VB, vascular bundle of leaf. Following one-way analysis of variance, different letters (e.g. a, b, c, etc.) between groups indicate statistically significant differences (*P* < 0.05), whereas identical letters denote insignificant differences among corresponding groups. (C) Heatmap of marker gene expression for all clusters (top 5). Note: Legend values represent the expression abundance of a gene, with larger values indicating a higher abundance of gene expression in that cell and vice versa.

To ensure the accuracy of cell type annotation results, tea leaf tissues were initially separated into vascular bundle, leaf mesophyll, lower epidermis, and trichome tissues (Fig. 4A), and qRT-PCR was utilized to detect the expression patterns of marker genes for different cell types in these four tissues. The findings, depicted in Fig. 4B, revealed consistent expression patterns of these marker genes across the qRT-PCR results from the four leaf tissues and the scRNA-seq data. This confirmation underscores the high accuracy of cell type identification. Additionally, previous studies have reported that the PSBW gene [16] is involved in maintaining the stability of Photosystem II (PS-II) to ensure normal light and action in chloroplasts. As palisade tissue is a crucial component of leaf mesophyll tissue and a major site for light and action, it contains a large number of chloroplasts. Therefore, the high expression of the PSBW gene in leaf mesophyll tissue further supports its functional role in influencing light and action genes, emphasizing the accuracy of cell type identification in this experiment.

We also selected the top five genes with the highest expression abundance in each cluster to generate a heatmap (Fig. 4C and Table S14). These genes can serve as novel marker genes for cell types within the respective clusters in tea plants. Notably, during this process, we identified, for the first time, 35 marker genes specific to the trichome clusters (Table S14). The discovery of these genes will contribute to more accurate identification of trichome cells in future single-cell sequencing studies of tea plants.

### Cell cycle regulation during trichome development

**Figure 5:**
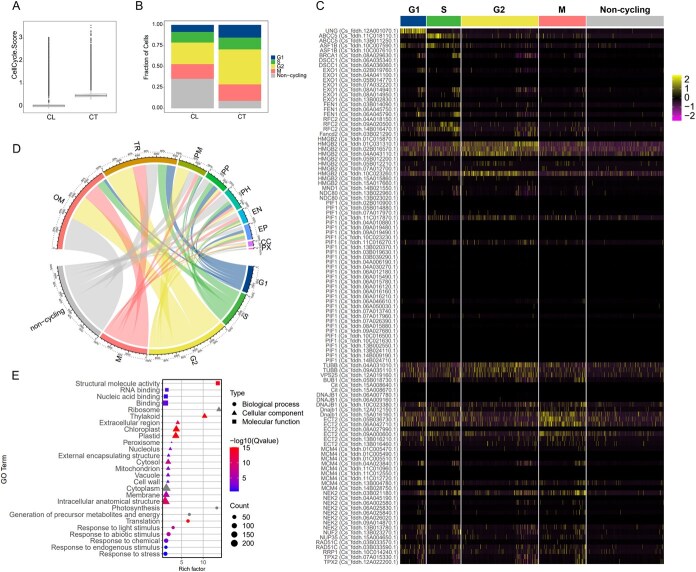
Cell cycle analysis of single-cell transcriptomes. (A) Cell cycle scores for different samples. (B) Percentage stacked plot of the number of cells with different cell cycles for different samples. (C) Heatmap of cyclin gene expression in different cell cycles. Note: Legend values represent the expression abundance of a gene, with larger values indicating a higher abundance of gene expression in that cell and vice versa. (D) Circos plot illustrating the relationship between different cell types and corresponding cell cycle stages. (E) GO enrichment of significantly upregulated genes in G2 phase cells. Note: Gray color indicates Q-values approaching zero.

Tea plant trichomes are specialized single-cell structures derived from epidermal cells, belonging to nonsecretory glandular hairs [6]. To gain deeper insights into the molecular mechanisms governing trichome development, we initially assessed cell cycle phases by examining cyclin expression, providing a visual representation of cellular differentiation dynamics in each sample. During each cell cycle, distinct cyclins exhibit specific expression patterns. Evaluating the expression of these characteristic cyclins for each cell cycle phase [17], we can assign scores to assess the cell in terms of its corresponding cell cycle phases. The cell cycle associated with the highest score is regarded as the cell’s current cell cycle phase. Conversely, if scores for the corresponding cell cycle phase were all <0, it indicated that the cell was not in that particular phase. Upon comparison with the CL group, we found that the CT group exhibited a higher overall score in the cell cycle (Fig. 5A), indicating a more active differentiation and proliferation characteristic in the cells of the CT group. Across all samples, we detected four major cell cycle phases: G1 phase (preparation for DNA synthesis), S phase (DNA synthesis phase), G2 phase (post-DNA synthesis phase), and M phase (mitotic phase). It is noteworthy that the proportion of cells in the M phase did not differ significantly between the two samples; however, the CT group exhibited a significantly higher proportion of cells in the G2 phase compared to the CL group (36.3% vs. 28.1%) (Fig. 5B). To further elucidate the expression patterns of cyclin protein genes in different cell cycle stages, we utilized a heatmap for visualization (Fig. 5C). The heatmap results clearly demonstrate a trend of concentrated expression of characteristic cyclin protein genes in their respective periods, confirming the accuracy of our cell cycle inference results. Additionally, we conducted statistical analysis on the distribution of cell cycles in different cell types. The results showed that the proportion of cells in the G2 phase in the trichome cell cluster was as high as 37.5% (Fig. 5D). This finding is consistent with previous research reports [18], indicating a close correlation between the initiation of leaf trichome development and changes in the cell cycle, especially the transition of epidermal cells from mitosis to endoreplication. Interestingly, a substantial distribution of G2-phase cells was also observed in the mesophyll cell cluster, particularly within the palisade tissue cluster, where they accounted for 45.8% (Fig. 5D). We speculate that this may be due to both palisade tissue and trichome cells belonging to specialized cell types, requiring endoreplication to complete their differentiation processes.

**Figure 6:**
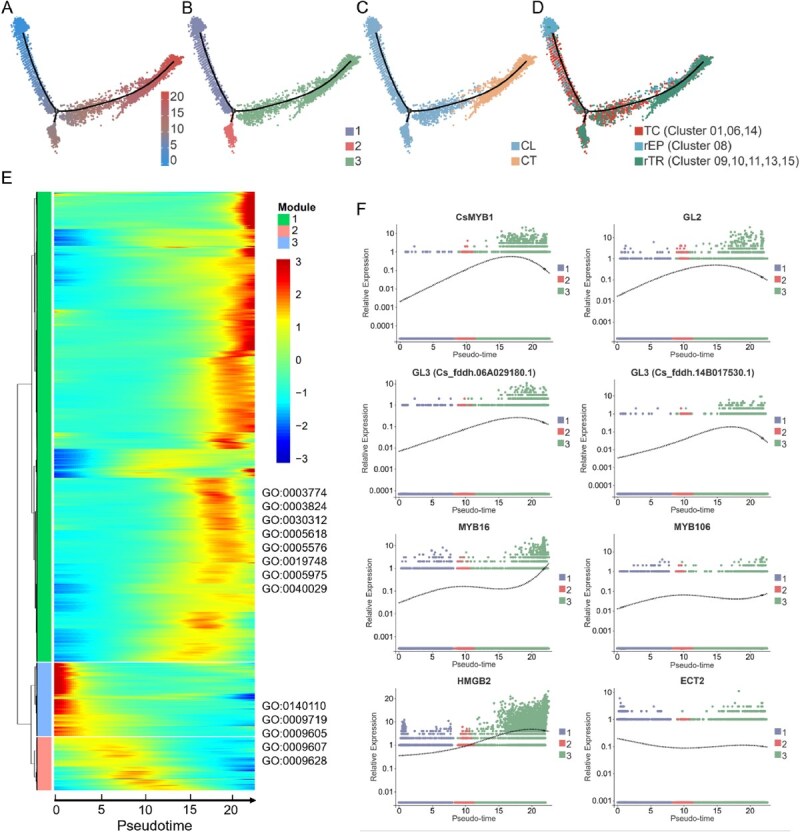
Pseudotime trajectory analysis of trichome cell subpopulations. (A) Cell scores along the pseudotime trajectory. (B) Different stages of pseudotime trajectory, classified based on cell scores. (C) Various samples along the pseudotime trajectory. (D) Distinct cell subpopulations along the pseudotime trajectory. (E) Heatmap of differentially expressed genes across the pseudotime axis coupled with GO functional annotations. Note: Legend values represent the expression abundance of a gene, with larger values indicating a higher abundance of gene expression in that cell and vice versa. (F) Scatter plot of expression trends for trichome development-related genes across the pseudotime trajectory. Note: Different colors indicate the states of pseudotime. Each dot represents a cell. TC indicates transitional cell clusters; rEP, reselected epidermal cell clusters; rTR, reselected trichome cell clusters.

Subsequently, we screened for genes upregulated in different cell cycle phases (Fig. S5A), successfully identifying 1309 genes with significant differences. Furthermore, through detailed bubble plot visualization (Fig. S5B), we further revealed the molecular expression profiles of cells at various cell cycle phases. Based on this, we selected the top 5 significantly upregulated genes and established them as novel cycle protein feature genes, aiming to provide a solid reference for the accurate identification of plant cell cycles in the future. During the study, we focused on the UMAP plot of G2 phase feature protein genes (Fig. S5C–G). Interestingly, these newly discovered feature genes are mainly concentrated in the trichome cell subpopulation, with additional distribution in mesophyll cells and epidermis. Previous literature on *A. thaliana* has reported that the transcription level of HTR-2 is higher in cells from S to G2 phase, and its promoter can form a cell cycle fluorescent marker with the CDT1a protein [19]. Further analysis showed that significantly upregulated genes in the G2 phase exhibit diverse functional activities in GO annotations, including structural molecular activity (GO:0005198), RNA binding (GO:0003723), ribosome (GO:0005840), and precursor metabolite and energy generation (GO:0006091), among others (Fig. 5E). These cells not only demonstrate high functional activity but also respond rapidly to various external stimuli. In summary, our research results strongly support the important role of the G2 phase in the formation and development of trichome cells in tea plant leaves.

### Pseudotime trajectory analysis of trichome development

Tea plant trichomes predominantly develop on the lower epidermis (LE) of the leaf [20]. A comparable ratio of cells derived from CL and CT samples was observed in cell clusters 1, 6, and 14, with CL/CT ratios of 0.93, 1.65, and 1.49, respectively (Fig. 2C). These clusters also showed high co-expression of marker genes associated with both trichomes and epidermis (Fig. S6), indicating that the cells are likely in a transitional state between epidermal and trichome lineages. Based on these findings, we reclassified clusters 1, 6, and 14 as transitional cell (TC) clusters. Following the exclusion of TCs, the remaining EP and TR clusters were subsequently redefined as reselected epidermal (rEP, cluster 8) and trichome cell clusters (rTR, clusters 9, 10, 11, 13, and 15). Subsequently, we performed pseudotime analysis using monocle2 software on the TC, rEP, and rTR clusters. Through this analysis method, we aim to elucidate more clearly the dynamic changes of each cell cluster during trichome development and their molecular regulatory networks. Considering the biological significance of the samples, we set the EP cell cluster as the starting point (Fig. 6A). Pseudotime analysis indicated one branching point in the pseudotime trajectory, dividing all cells into three states (Fig. 6B). State 1 and State 2 were primarily composed of CL samples, whereas State 3 (the trajectory endpoint) was predominantly composed of CT samples (Fig. 6C). It can be inferred that the predominant accumulation at the end of state 3 is trichome cells. Additionally, Fig. 6D shows that in the first stage, rEP clusters are the majority, while rTR clusters are mainly distributed in states 2 and 3, and TC clusters are evenly distributed throughout the process. In summary, epidermal cells eventually differentiate into trichome cells after undergoing a critical differentiation step. Moreover, based on the analysis results of the pseudotime trajectory, we constructed a heatmap of differentially expressed genes during trichome development (Fig. 6E) and categorized them into three modules based on their expression patterns. Genes in module 1 showed significant upregulation mainly at the end of the pseudotime trajectory (state 3), with functions primarily involved in cell morphology construction. Genes in modules 2 and 3 exhibited noticeable increases in the early (state 1) and middle (state 2) stages, and their functional annotations indicated their sensitivity to various stimuli and strong developmental capabilities, aligning well with the characteristics of plant epidermal cells [21].

Next, we further filtered out the top 100 genes with the most significant differences in the heatmap (Fig. S7), aiming to pinpoint more precisely the key genes closely associated with trichome development. Among these genes are several homologous genes known from existing literature to be related to trichome development [22, 23]. We delved deeper into the expression patterns of these genes along the developmental trajectory (Fig. 6F). Of particular note, four genes, *GL2* (*Cs_fddh.02B030590.1*), *CsGL3* (*Cs_fddh.14B017530.1*), *GL3* (*Cs_fddh.06A029180.1*), and *CsMYB1* (*Cs_fddh.08A016340.1*), exhibit a gradually increasing expression trend in the late stages of the developmental trajectory. To verify the reliability of these findings, we employed RT-PCR technology to measure the actual expression levels of these key genes in trichome and epidermal cells. The results showed a high consistency between the bulk RNA fluorescence quantification data for these genes and the scRNA-seq results (Fig. S8), thereby validating the accuracy of the single-cell sequencing data.

**Figure 7:**
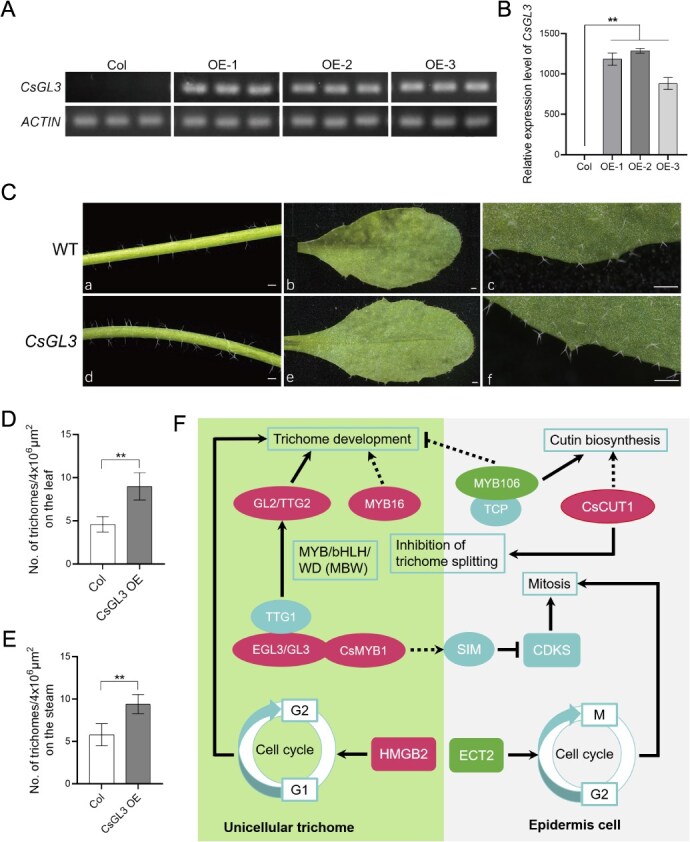
Functional characterization of the *CsGL3* gene and developmental analysis of *C. sinensis* trichomes. (A) RT-PCR analysis of *CsGL3* expression in *CsGL3-*overexpression (*CsGL3-*OE) lines*.* (B) qRT-PCR analysis of *CsGL3* transcript levels in *CsGL3-*OE lines. (C) Phenotypic observation of leaves from *CsGL3*-OE transgenic plants. Bar = 500 μm. (D) Quantification of trichome density on leaves of *CsGL3-*OE lines (*n* = 3). (E) Quantification of trichome density on stems of *CsGL3-*OE lines (*n* = 3). (F) The model illustrating the role of *CsGL3* in trichome development. Statistically significant differences from control were determined by Student’s *t*-test: ^*^P < 0.05; ^**^P < 0.01. Values are means ± SEM of three biological replicates.

In tea plants, scatter plots depicting cell developmental trajectories show that the expression level of *CsGL3* gradually increases during trichome development (Fig. 6F). These transcriptional trajectories, representing dynamic changes in gene expression patterns along pseudotime, provide a high-resolution view of the regulatory transitions during cellular differentiation. Using the floral dip method, we overexpressed the *CsGL3* gene in *Arabidopsis* (Fig. 7A and B). Compared to the wild-type plants, the transgenic *Arabidopsis* mutants exhibited a significant increase in trichome density on both stems and leaves (*P* < 0.01) (Fig. 7C–E). These results indicate that *CsGL3* plays an important role in trichome development in tea plants. Previous studies have demonstrated that GL3 is a crucial gene involved in plant development, encoding a basic helix–loop–helix (bHLH) transcription factor. In *Arabidopsis*, GL3 interacts with GL1 and TTG1 to form the MYB-bHLH-WD40 (MBW) complex, a key regulatory module that activates the expression of trichome initiation genes such as *GL2* [24] (Fig. 7F). Moreover, the formation and function of the MBW complex are conserved across plant species, suggesting its broader role beyond *Arabidopsis*. For example, in rice, OsGL3B interacts with OsGL1E and OsTTG1A, highlighting its conserved functionality in trichome-related processes across diverse plant taxa [23].

Furthermore, to investigate the practical effects of these genes on trichome development and to verify the reliability of the analysis results, this study randomly selected several members from these key candidate genes for transgenic functional validation in the model plant *A. thaliana*. Interestingly, we also identified a homologous sequence of the *CUT1* gene in the tea plant, designated as *CsCUT1 (Cs_fddh.02B010090.1)*. This gene family has been reported in other species to encode the very-long-chain fatty acid condensing enzyme, which plays a crucial role in the biosynthesis of leaf cuticular layers [25, 26]. This gene exhibits regulatory effects that inhibit trichome branching during trichome development. In tea plants, scatter plot maps depicting cell developmental trajectories show that the expression level of the *CsCUT1* gene significantly increases in the early to mid-stages of trichome development (states 1–2) and gradually decreases in the later stages (states 2–3) (Fig. S9A). In *CsCUT1* overexpressing *Arabidopsis* mutants (Fig. S9B–D), trichome branching is significantly reduced. Additionally, *CsCUT1* overexpression also led to a 57% decrease in trichome density compared to wild-type plants (*P* < 0.01) (Fig. S9E), suggesting a potential link between cuticle biosynthesis and trichome development in tea leaves, similar to that observed in tomato and rice [27, 28]. Typically, tea plant trichomes are unicellular and unbranched in morphology [6, 29], which is consistent with the phenotypes observed in *CsCUT1* overexpressing mutants. This demonstrates that *CsCUT1* plays a crucial role in the early stages of trichome development by reducing trichome branching. In summary (Fig. 7F), by screening differentially expressed genes and analyzing their expression patterns during trichome development, this study not only confirms the significant regulatory potential of these genes in trichome development but also reveals the complex interactive mechanisms of positive and negative regulation during this process.

### The regulatory mechanism of epidermal cell differentiation into trichomes and guard cells in tea plant leaves

**Figure 8:**
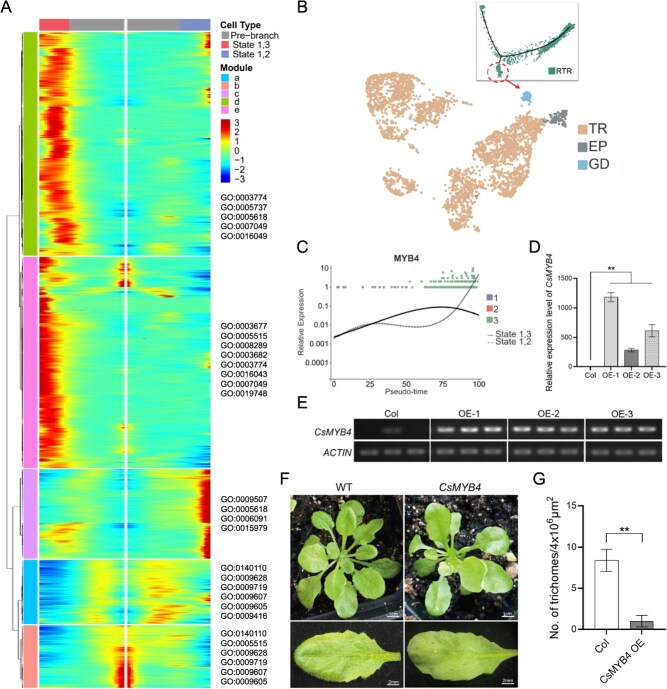
Analysis of the developmental regulation of trichome cells and guard cells in *C. sinensis* leaves. (A) Heatmap of differentially expressed genes associated with cell differentiation fate and their GO functional annotations. Note: Legend values represent the expression abundance of a gene, with larger values indicating a higher abundance of gene expression in that cell and vice versa. (B) Schematic representation of the pseudotime trajectory for the development and differentiation of GD. (C) Scatter plot of expression patterns for *MYB4* associated with cell differentiation fates. Note: Different colors indicate the states of pseudotime. Each dot represents a cell. (D) qRT-PCR analysis of *CsMYB4* transcript levels in *CsMYB4-*overexpression (*CsMYB4-*OE) lines. (E) RT-PCR analysis of *CsMYB4* expression in *CsMYB4-*OE lines*.* (F) Phenotypic observation of leaves (bar = 2 mm) from *CsMYB4*-OE transgenic plants (bar = 1 cm). (G) Quantification of trichome density on leaves of *CsMYB4*-OE lines (*n* = 3). Statistically significant differences from control were determined by Student’s *t*-test: ^*^P < 0.05; ^**^P < 0.01. Values are means ± SEM of three biological replicates.

The results of the aforementioned pseudotime analysis indicate that epidermal cells undergo a significant differentiation process before ultimately developing into trichome cells (Fig. 6B). To comprehensively elucidate the molecular mechanisms influencing cell fate differentiation, we applied Branch Expression Analysis Modeling (BEAM) on the basis of trichome development trajectories to generate a differential gene expression landscape of cell fate differentiation. As depicted in Fig. 8A, these genes were clustered into five modules. During the transition from state 1 to state 3 (Figs 6A and 8A), the expression levels of genes in modules d and e significantly increased, with their functions primarily focusing on cellular cytoskeletal dynamics, cell cycle regulation, and growth. Trichome cells on leaf surfaces generally possess highly specialized cellular structures, thus requiring extensive cellular morphogenesis before epidermal cells can differentiate into trichome cells. Evidently, the enriched gene functions align well with this pattern.

It is worth noting that in this study (Figs 6D and 8A), cells of the rTR cluster predominantly clustered at the end of state 2 and state 3 in the pseudotemporal trajectory. Moreover, in the cell fate map from state 1 to state 2, the functional annotations of module c genes, which showed a significant upregulation trend, suggest their involvement in chloroplast formation and photosynthesis. Trichomes on tea leaves, like those in *Arabidopsis*, are nonglandular trichomes and typically lack chloroplasts [12]. Therefore, this result demonstrates that before epidermal cells develop into trichome cells, some cells differentiate into other types of cells capable of participating in light and photosynthesis. While these cell types may be spatially close to trichome cells on the leaf surface (difficult to discern with previous dimensional clustering analysis), they exhibit distinct physiological functions. And the distribution of cells from the rTR cluster along the developmental trajectory suggests that these cells also exist within the rTR cluster (Fig. 6D). Consequently, we performed a redimensionality reduction and clustering analysis of cells from the rTR cluster, resulting in the identification of 10 new cell subgroups (Fig. S10A). Subsequently, employing the same methodology as previously described, a comparison of the marker genes of each cell cluster with a public database was conducted. This analysis led to the identification of three types of cells: TR, guard cells (GD), and EP (Fig. S10B and C). The majority of GD clusters are clustered in the second stage (state 2) of the pseudotemporal trajectory (Fig. S10D). Additionally, we observed that GDs are primarily concentrated in the CL samples and are very rare in the CT samples (Fig. S10E). Therefore, the reason why the previous dimensional clustering analysis grouped GD and TR into the same cluster may be because both types are closely located morphologically, residing in the lower epidermis of leaves and originating from epidermal cells. However, guard cells possess intact chloroplasts and can participate in respiration and photosynthesis, enabling their differentiation from trichome cells in the analysis of pseudotime developmental trajectories. So, it can be inferred that during the process of epidermal cell differentiation into trichome cells, some epidermal cells concurrently differentiate into guard cells (Fig. 8B).

To further elucidate the regulatory mechanisms underlying epidermal cell fate differentiation, we proceeded to analyze the expression patterns of the top 100 most significantly differentially expressed transcription factors (TFs) implicated in this process. As depicted in Fig. S11, our analysis disclosed a discernible and contrasting expression pattern among these TFs along the developmental trajectories of states 1–3 (representing trichome development) and states 1–2 (corresponding to guard cell development). Significantly, genes like *MYB17*, *WER*, and *ROC2* were found to be strikingly upregulated during the progression of trichome development, whereas they displayed an opposite trend during the course of guard cell development, as shown in Fig. S12. However, the expression dynamics of the *MYC2* gene seem particularly favorable for directing epidermal cells toward the differentiation into guard cells. In sum, these distinctive expression profiles of the selected TFs collectively unravel a sophisticated regulatory network that orchestrates the intricate decision-making process by which epidermal cells differentiate into either trichome or guard cells during their developmental trajectory.

Additionally, we identified a novel member of the *MYB* family from these key candidate transcription factors (TFs)-*CsMYB4 (Cs_fddh.08A016340.1)*. This transcription factor contains a specific SANT domain and exhibits significant negative regulatory effects during trichome development. Comprehensive phenotypic analysis of CsMYB4-overexpressing *Arabidopsis* lines demonstrated a strong inhibitory effect on trichome formation, with trichome density reduced by 88% in leaves (*P* < 0.01) compared to wild-type plants. In tea plants, scatter plot maps depicting cell fate determination and differentiation processes show that the expression level of the *CsMYB4* gene gradually increases during the transition of epidermal cells to guard cells (states 1–2), whereas it shows a decreasing trend in the later stages of epidermal cells transitioning to trichome cells (states 1–3) (Fig. 8C). Results from genetic transformation experiments (Fig. 8D–G) indicate that in *CsMYB4* overexpressing *Arabidopsis* mutants, trichome development is significantly inhibited, displaying abnormal trichome phenotypes. Therefore, *CsMYB4* not only has a significant inhibitory effect on trichome cell development but may also play a positive role in guard cell development. In summary, the unique expression patterns of these transcription factors collectively reveal a sophisticated regulatory network that orchestrates the complex decision-making process of epidermal cells differentiating into trichome or guard cells during development.

## Discussion

### Completion of the first chromosome-level assembly of a triploid tea plant genome

The *C. sinensis* FDDH, a primary cultivar for white tea production, is a naturally hybridized triploid tea plant. Given the substantial reliance of polyploidy research on a high-quality genome, our study’s assembly of the homologous triploid genome fills a significant gap in the field of tea research. Notably, the integration of high-fidelity long reads with Hi-C scaffolding effectively overcame common challenges in polyploid genome assembly, such as haplotype phasing ambiguity and sequence redundancy. The PacBio HiFi technology, with its exceptional accuracy and long reads, has demonstrated remarkable efficacy in resolving complex polyploid genomes, as evidenced by successful applications in autotetraploid sugarcane [30], autotetraploid alfalfa [31], and octoploid strawberry [32]. In the present study, we generated high-depth PacBio HiFi data for the triploid Fuding Dahaocha genome and further integrated the ALLHiC algorithm [33], previously developed by our group, with manual curation. This combined strategy has effectively minimized assembly redundancy and enhanced the accuracy of haplotype phasing, thereby robustly addressing the key challenges associated with triploid genome assembly and resulting in a highly contiguous and accurate representation of the three homologous chromosome sets. Moreover, we employed a method of distinguishing chromosome-specific k-mers for analysis, thereby identifying the homologous characteristics of the three sets of chromosomes in *C. sinensis* FDDH (Fig. 1D). Additionally, the SNP-informed maximum likelihood phylogenetic analysis (Fig. S3) further corroborates this by revealing exceptionally conserved evolutionary relatedness among the three chromosome groups, underscoring the homologous nature of this important tea variety’s genome structure. This finding also suggests that the multi-trichome trait of *C. sinensis* FDDH, compared to diploid tea plants, is more likely influenced by gene dosage effects [34].

### Construction of a single-cell atlas of trichomes in tea plant leaves

Currently, there is a paucity of literature on single-cell atlases for woody plant leaves. The main challenges are twofold: Firstly, some marker genes used in model plants do not fully translate to woody species, leading to uncertainties in the precise annotation of cell types. Secondly, the abundant secondary metabolites in woody plants, particularly the tea plant, pose significant impediments to protoplast isolation. Moreover, conventional methods for protoplast extraction struggle to isolate trichome cells from leaf tissues. To address this challenge, we employed single-nucleus sequencing technology to tackle the issue of acquiring transcriptional data from trichome cells. Given the lack of a stable and efficient genetic transformation system for tea plant and the interference from secondary metabolites that result in high autofluorescence backgrounds *in situ* hybridization experiments, we adopted the methodology outlined by Wang *et al.* [2] to validate four tissue types—TR (trichomes), LM (laminar mesophyll), LE (leaf epidermis), and VB (vascular bundle)—in tea leaves. This approach yielded 86 novel, reliable marker genes for these tissues, including 35 markers specifically related to trichomes (Fig. 4C).

Trichomes are minute hair-like structures on plant leaf surfaces that play critical roles in plant growth and environmental adaptation. Field observations have consistently shown that the dense trichome coverage in tea plants contributes to its enhanced resistance against piercing-sucking pests like tea green leafhoppers, while also reducing water loss under drought conditions [35]. The *GUX2* gene, encoding a xylan synthesis-related protein, potentially participates in cell wall synthesis and remodeling within leaf trichomes, thereby influencing their morphology and function [36]. Furthermore, the *CUT1* gene encodes a protein involved in the biosynthesis of cuticular wax in epidermal cells, and its deficiency has been shown to affect trichome morphology and functionality [37]. In this study, we observed that overexpression of the *CsCUT1* gene inhibited trichome branching in *Arabidopsis* leaves. Given that trichomes in tea plants are uniformly unbranched, *CsCUT1* may play a pivotal role in determining the morphology of tea leaf trichomes. In *Arabidopsis*, trichome branching is driven by the polarized growth of epidermal cells, primarily regulated through apical and basal polarity mechanisms. Based on these observations, we hypothesize that *CsCUT1* might contribute to the regulation of trichome cell polarity in tea leaves, a hypothesis that warrants further exploration. Based on these observations, we hypothesize that CUT1 homologs may regulate trichome cell polarity in tea plants, potentially due to their conserved role in plant trichome development. Future efforts employing tea protoplast systems will be essential to validate the functions of candidate genes within the native polyploid context of tea.

Thus, trichome-specific marker genes provide valuable insights into the identification of trichome cells within leaves and the underlying developmental mechanisms, thereby enhancing our understanding of plant leaf development and adaptive strategies. It should be noted, however, that this study provides a high-resolution snapshot of trichome development at a specific leaf stage, yet it does not capture temporal dynamics across bud-to-leaf maturation. Additionally, while our snRNA-seq analysis focused on leaf tissues, trichome development may be influenced by systemic signals from other organs such as stems and buds. Future multi-tissue single-cell studies would help elucidate such inter-organ regulatory networks. Future time-series snRNA-seq studies will be essential to unravel the stage-specific regulatory programs underlying trichome development.

### The impact of the cell cycle and differentiation on trichome development

Trichomes on plant leaves are derived from epidermal layer cells and typically undergo three distinct stages: initiation induction, endoreduplication, and expansion and morphogenesis. As other epidermal cells continue to progress through the mitotic cycle, trichome precursor cells commit to their cellular fate and cease the mitotic cell cycle, transitioning instead into a phase of endoreduplication characterized by chromosome multiplication. In this study, we employed expression patterns of cell cycle proteins to assess the differentiation activity in cells across different samples, revealing that CT samples exhibit significantly higher overall cell cycle scores compared to CL samples, indicative of more active cell proliferation and differentiation in CT samples. This observation aligns with the biological process underlying leaf trichome development, where robust cell proliferation and differentiation are critical for trichome formation.

Notably, while no significant difference was observed in the proportion of M-phase cells between the two samples, G2-phase cells were significantly more prevalent in CT samples compared to CL samples. Furthermore, within the subpopulation of trichome cells, G2-phase cells were particularly prominent, suggesting a potentially pivotal role for the G2-phase in trichome development. The onset of trichome development is tightly coupled with alterations in the cell cycle, notably the transition of epidermal cells from mitosis to endoreduplication. Moreover, our research identified 20 significantly differentially expressed marker genes that provide a robust reference framework for accurate identification of plant cell cycle phases in future studies. Moreover, we detected contrasting expression patterns of two genes closely related to the cell cycle—High Mobility Group Box Protein B2 (HMGB2, *Cs_fddh.01C031310.1*) [38] and YTH Domain Family Protein (ECT2, *Cs_fddh.05B036730.1*) [39, 40]—during trichome development. This finding provides further evidence that alterations in the cell cycle have a substantial impact on trichome development.

During the process of trichome cell development, a subset of epidermal cells differentiates into guard cells with distinct characteristics. To unravel the regulatory mechanisms governing this cell fate decision in epidermal cells, we investigated the expression patterns of key transcription factors. Previous studies have shown that ectopic expression of *MYB17* can induce conical cells and promote direct differentiation of stamen epidermal cells into trichomes, emphasizing its pivotal role in trichome development. Concurrently, *WER* exhibits a similar expression pattern to *MYB17* and can rescue trichome developmental defects caused by *GL1* mutations in *Arabidopsis*. ROC2, a homologous domain protein of *GL2*, also plays a positive role in trichome formation, while *GL2* itself functions as a central regulator controlling precise epidermal cell differentiation for trichome cell initiation and maturation [22, 41]. Furthermore, our analysis has highlighted genes like *MYC2* that are associated with promoting guard cell development, given its high expression levels observed in *Arabidopsis* guard cells and the corresponding trend seen in our study, suggesting *MYC2*’s significant involvement in determining epidermal cell fate. Interestingly, this study identified a *MYB* family member, *CsMYB4*, as a negative regulator of trichome development in tea plants. This transcription factor significantly suppressed trichome formation in *Arabidopsis*, resulting in a ‘hairless’ phenotype. Given the rarity of clustered trichomes observed on tea leaves, it is hypothesized that, similar to other plants, tea plants possess an intrinsic mechanism to regulate the spatial arrangement of leaf trichome cells [42]. *CsMYB4* emerges as a key regulatory gene governing this mechanism. These findings significantly advance woody plant research at the single-cell level resolution while establishing a critical framework for understanding cellular differentiation during leaf maturation. Looking forward, future studies utilizing spatial transcriptomics could validate the physical co-localization of these transitional states within specific leaf epidermal niches, thereby adding crucial spatial context to the developmental pathways proposed here.

Beyond elucidating the molecular mechanisms of trichome development, our findings offer tangible insights for the genetic improvement of tea plants. The trichome-specific marker genes identified here, particularly those implicated in stress adaptation and specialized metabolism, provide valuable candidates for marker-assisted selection aimed at enhancing trichome density, which is closely associated with tea aroma and stress resilience in commercial varieties. However, our genomic and single-cell analyses were conducted primarily in a single triploid cultivar, which, while informative, may not fully represent the genetic diversity within the species. Future population-level genomic studies across diverse tea germplasm will be essential to assess allelic diversity, identify structural variations, and fully evaluate the breeding potential of trichome-related traits. While our study provides molecular evidence supporting the role of trichomes in stress adaptation, future field validation across different environments will be crucial to confirm the agronomic value of these traits. Furthermore, while this study provides genomic and transcriptomic insights into trichome development, it does not address the potential role of epigenetic regulation mechanisms such as DNA methylation and histone modifications. Future investigations incorporating epigenomic analyses will be essential to fully elucidate the regulatory landscape governing trichome development and polyploid genome stability in tea plants. Future studies incorporating diverse germplasm and developing robust tea-specific genetic tools will be essential to translate these molecular insights into practical breeding applications.

## Conclusion

This study presents three advancements in tea plant genomics and developmental biology. First, we report the first chromosome-level assembly of the triploid genome of *C. sinensis* FDDH, a key cultivar for white tea production. Utilizing chromosome-specific k-mer analysis and SNP-informed phylogenetics, we elucidated conserved synteny and evolutionary parallelism among the three homologous chromosome sets, establishing a genomic basis for triploidy-associated phenotypic divergence in *C. sinensis* FDDH. Second, we constructed the first single-cell atlas of trichomes in woody plant leaves by overcoming challenges in protoplast isolation and secondary metabolite interference. Through single-nucleus sequencing and marker validation, we identified 35 trichome-specific markers and functionally characterized *CsCUT1* as a regulator of trichome branching suppression, highlighting its role in shaping the unbranched trichome morphology unique to tea plants. Third, we explored cell cycle dynamics during trichome development, revealing that G2-phase dominance in trichome cells correlates with endoreduplication and differentiation. We further identified *CsMYB4* as a negative regulator of trichome formation, suggesting its potential role in spatial patterning. Collectively, these findings establish foundational genomic resources for polyploid tea research, advance single-cell methodologies for woody species, and unravel molecular mechanisms governing trichome development, offering transformative implications for tea genetic improvement and plant developmental biology.

## Materials and methods

### Whole genome sequencing and hi-C assisted assembly

The tea plant material of the cultivar (*C. sinensis* FDDH) was collected from its native habitat in Wangjiayang Village, Fuding County, Ningde City, Fujian Province, China. Roots containing active root apical meristem tissues were first collected from hydroponically grown seedlings and sent to OMIX Technologies Corporation (Chengdu, Sichuan, China). The genome sizes of *C. sinensis* FDDH, maize, and tomato were compared using the FACSCalibur cell sorting system (Becton Dickinson, USA) [43]. Subsequently, karyotype analysis of *C. sinensis* FDDH was performed by the same company using FISH to determine chromosome ploidy [44].

DNA extraction was performed using fresh young shoots of *C. sinensis* FDDH as the primary material. The high-quality DNA obtained was sent to Beijing Berry Genomics Technology Co., Ltd., for the construction of Circular Consensus Sequencing (CCS) (HIFI) libraries, Illumina libraries, and Hi-C libraries. Sequencing of the CCS library was conducted on the PacBio Sequel II platform, involving six SMRT cells, generating 332 Gbp of unfiltered raw reads. These raw reads were then corrected using the CCS mode to produce refined data, and preliminary assembly was performed using Canu (v1.9) [45]. For the Hi-C library, raw sequencing data were filtered using Trimmomatic (v0.33) [46] to remove low-quality reads and adapters. The filtered reads were then mapped to the preliminary contig-level genome using the Burrows-Wheeler Aligner (BWA) software [47]. Sequences located more than 500 bp from restriction enzyme cutting sites were removed using filtering scripts. The ALLHiC software [48] was employed to sort and anchor all contigs based on the intensity of interaction signals, resulting in 45 high-quality chromosome sequences and remaining unanchored contigs. Assembly integrity was evaluated using Core Eukaryotic Genes Mapping Approach (CEGMA) [49] and Benchmarking Universal Single-Copy Orthologs (BUSCOs) (v3.0.2, embrhyta_odb10) [50].

Additionally, following the methodology of Mitros *et al.* [51], we analyzed the clustering distribution pattern of 13-mer specific k-mers across the 45 chromosomes of *C. sinensis* FDDH. BWA was used to map Illumina second-generation short reads of *C. sinensis* FDDH to its assembled genome. Valid reads originating from the three haplotypes were selected. The second-generation sequencing data for each haplotype were mapped to the *C. sinensis* TGY genome using BWA, and high-quality chromosomal SNPs were identified using Genome Analysis Toolkit (GATK) software [52]. The SNPs obtained in this study were integrated with the variation data from 196 tea cultivars reported by Zhang *et al.* [15], and missing data were effectively filtered. A phylogenetic tree based on maximum likelihood was constructed using the TreeBeST tool (TreeBeST: Tree building guided by species tree http://treesoft.sourceforge.net/treebest.shtml).

### Genome annotation and evaluation

The *C. sinensis* FDDH genome was analyzed using RepeatModeler and RepeatMasker [44] to identify the overall distribution of repetitive sequences. These repetitive sequences were then masked to provide a foundation for the subsequent steps in gene structure prediction.

Gene model prediction for the *C. sinensis* FDDH genome was performed using the GETA (V2.6.1) (https://github.com/chenlianfu/geta) [53]. The main steps involved are as follows: First, transcriptomic data from the stems, leaves, flowers, and roots of *C. sinensis* were processed using Trimmomatic software [46] to remove low-quality reads and adapters, followed by mapping the filtered reads to the genome using HISAT2. Open reading frames (ORFs) were then predicted with TransDecoder (https://github.com/TransDecoder/) [54] to generate an initial gene model. Second, a homologous protein sequence database was constructed, and the gene models from step 1 were matched against this database using Genewise [55] for homologous gene prediction. Third, the results from the first two steps were integrated and further refined using the AUGUSTUS software (http://augustus.gobics.de) [56, 57] for enhanced gene prediction. Finally, the predictions were filtered using the PFAM database (http://pfam.xfam.org/) to obtain a high-quality genome model. Additionally, the *C. sinensis* FDDH proteome was queried against the Plant Transcription Factor Database (PlantTFDB) (https://planttfdb.gao-lab.org/) [58] to identify transcription factors (TFs). To visualize the genomic features of *C. sinensis* FDDH, including gene density, GC content, gene expression, repeat sequence density, and synteny information, Circos (v0.69) [59] was used.

### Preparation of protoplasts from tea leaves and trichome cell nucleus suspensions

The protoplast extraction method, primarily derived from the study conducted by Wang *et al.* [1, 2] with some adaptations, involves the following steps. Initially, the protoplast buffer was prepared, comprising 5 M NaCl, 0.5 M CaCl_2_, 1 M CaCl_2_, 1 M MgCl_2_ (6H_2_O), 2 M KCl, 0.2 M MES, and 0.8 M mannitol, with adjustments made using ultrapure water. Subsequently, digestive enzymes (1%–1.5% Cellulase R10, 0.5% Pectolyase, 0.2%–0.4% Macerozyme R10) were added to the protoplast buffer, forming an enzyme solution, which was stored in the dark. Following this, three to four tea tree leaves were finely chopped using a sharp blade and thoroughly immersed in the enzyme solution (biological replicates from three independent plants). A vacuum was then applied for 30 min. The other steps were not significantly changed.

Due to the inefficiency of conventional protoplast extraction method in isolating trichomes from tea plant leaves (biological replicates from three independent plants), we employed a combined strategy involving nuclear extraction followed by flow cytometry sorting to precisely isolate high-quality nuclei for library construction [60, 61]. This sorting step was applied solely for the preparation of sequencing libraries and not during the downstream bioinformatic analysis. Finally, scRNA-seq was performed on the 10× Genomics platform.

### Library preparation and sequencing for scRNA-seq

The high-throughput sequencing for this study was conducted by Guangzhou GeneDenovo Biotechnology Co., Ltd. (China), using the 10× Genomics platform. The fundamental principle behind the preparation of single-cell transcriptome libraries on the 10× Genomics platform involves encapsulating individual cells within isolated droplets using the Chromium™ microfluidic system. This process uniquely labels the cells with barcodes, enabling the differentiation of transcripts from distinct cell sources. Following library preparation, rigorous quality control procedures were implemented, and sequencing was performed using the Illumina NovaSeq 6000 platform with PE150 sequencing [62].

### Data preparation and quantitative expression analysis in scRNA-seq

In Illumina paired-end sequencing, Read1 consists of a 16-bp GemCode barcode (used to distinguish different cells) and a 10-bp unique molecular identifier (UMI), which uniquely tags each RNA molecule. Read2 contains cDNA sequence fragments. The Cell Ranger software (https://www.10xgenomics.com/cn/support/software/cell-ranger/), utilizing the Spliced Transcripts Alignment to a Reference (STAR) aligner, was employed to align Read2 sequences to the reference genome (the *C. sinensis* FDDH genome). Based on GTF annotations, the alignment results were classified into exonic, intronic, or intergenic regions. Cell Ranger further aligned exonic reads to the annotated transcripts, identifying transcript reads and selecting uniquely mapped reads for UMI counting.

Following this, Cell Ranger was used to filter and correct barcodes and UMIs, retaining only validated, effective barcodes and UMIs for UMI counting. Redundancies were removed for each barcode corresponding to a specific gene ID, with the unique UMI count serving as a measure of gene expression level in individual cells. Assuming an expected cell count of *N*, barcodes were ranked in descending order of UMI count, and the top N barcodes were selected. The UMI count corresponding to the 99th percentile was denoted as m, and barcodes with UMI counts less than m multiplied by 10% were filtered out, while others were considered valid cells. Additionally, the EmptyDrops method was applied to further identify and retain cells with lower RNA content.

We performed cell quality control using the Seurat package (https://github.com/satijalab/seurat) in R, which incorporated DoubletFinder for doublet identification and removal. The primary filtering criteria included the following: (a) Gene count per cell: 200–8000. Excessively high counts may indicate multiple cells within a single GEM. (b) UMI count per cell: <50 000. Elevated UMI counts suggest possible cell multipleting or contamination. Barcodes failing these thresholds were excluded to ensure data reliability. After quality control and cell filtering, the gene expression data were normalized and subsequently integrated using the Harmony method to correct for batch effects.

### Visualization and clustering of data

Seurat was used to perform cell clustering through principal component analysis (PCA), where each principal component represents a set of genes exhibiting similar expression patterns. Resampling tests were subsequently applied to evaluate the impact of each principal component on the single-cell dataset. Components with the highest enrichment and lower *P* values were selectively chosen, indicating their significant influence on the subsequent single-cell data analysis [42]. Following this, Seurat employed a graph-based clustering method to group the cells.

To reduce dimensionality and visualize the data, Seurat applied UMAP [63] to map the high-dimensional cell data into a two-dimensional space. Differential gene expression within each cell cluster was analyzed using Seurat’s rank-based test, identifying genes that were upregulated in each cluster [64]. The criteria for selecting differentially expressed genes included a log2 fold change (log2FC) ≥0.36, indicating an upregulation factor ≥ 1.28; a *P* value ≤0.01; and the requirement that the gene must be expressed in at least 25% of the cells within the target cluster.

### Identification of cell types

To delineate cell types in the leaves of *C. sinensis* FDDH, we conducted a direct homologous gene comparison between upregulated genes in clusters and established marker genes in *Arabidopsis*. The roster of marker genes was sourced from the Plant Cell Marker Database (PCMDB, http://www.tobaccodb.org/pcmdb/) [65]. Tea plant protein sequences were derived from the annotation of our assembled data, while *Arabidopsis* protein sequences were retrieved from TAIR. Using Blastp software (e-value: 1 − e5) and employing *Arabidopsis* marker genes as query sequences, we conducted a search for homologous genes in tea plants to discern upregulated marker genes. Cell cluster annotation scores were calculated based on three key metrics: the quantity of upregulated marker genes (weight: 20%), the magnitude of expression fold (weight: 50%), and the proportion of cells expressing these markers (weight: 30%). A higher score indicates a greater likelihood of association with the corresponding *Arabidopsis* cell type, whereas a lower score suggests a reduced likelihood. Ultimately, the cluster with the highest score was provisionally annotated as the corresponding *Arabidopsis* cell type.

Subsequently, following the method of Wang *et al.* [1, 2], we performed a simple separation of tea leaf tissues to obtain vascular bundles, mesophyll, abaxial epidermis, and trichomes. Homologous genes in tea plants were then precisely identified using *Arabidopsis* marker genes as queries. Real-time quantitative PCR (qRT-PCR) was employed to quantify their expression levels across the four tissue types. Finally, the *Arabidopsis* reference marker genes and qRT-PCR data were integrated to refine the assignment of specific cell types associated with each cluster.

### Cell cycle analysis and pseudotime trajectory analysis

In this study, we employed the Seurat R package to calculate cell cycle scores for each cell based on the expression levels of specific marker genes [17] associated with different cell cycle phases. The highest scoring cycle was selected to determine the cell’s position in the cell cycle stage. Cells with scores below 0.3 for all cell cycle phases were classified as noncycling cells [66].

We performed pseudotime trajectory analysis of cells in tea plant leaves using Monocle2 [67] software. This analytical approach assumes that cell differentiation is a process characterized by continuous changes in gene expression. It unveils the positional relationships of cells along the differentiation time axis by comparing the similarity of gene expression among cells. In this process, Monocle2 initially applies PCA for dimensionality reduction and subsequently utilizes reversed graph embedding to obtain the final trajectory information. Gene Ontology enrichment analysis of the selected genes was performed using the OmicShare platform (www.omicshare.com/tools).

### Vector construction, *Arabidopsis* stable transformation, and phenotype analysis

Target genes were amplified and cloned into the pCAMBIA1300 backbone vector under the control of *UBQ* promoters according to the previous study [68]. For *Arabidopsis* transgenic lines, *Agrobacterium tumefaciens* strain *GV3101* containing vectors was used for transformation of *Arabidopsis* plants by the floral dip method described previously [69]. The transgenic seedlings were grown in a growth chamber at 23°C under a 16-h light/8-h dark cycle and 60% relative humidity for further genotyping and phenotype analysis. The plant tissue samples were observed with a Nikon SMZ1270 microscope. Quantitative measurements were obtained from three independent lines with multiple biological replicates to ensure statistical robustness.

### RNA extraction and qRT-PCR

Leaf tissues of transgenic *Arabidopsis* plants were collected for total RNA extraction using TRIzol (Invitrogen, Carlsbad, USA) according to the manufacturer’s instructions. All experiments were carried out with three biological replicates. *Arabidopsis* cDNA was synthesized from total RNA using the TransScript All-in-One First-Strand cDNA Synthesis SuperMix (Transgen Biotech, Beijing, China), and qRT-PCR was performed using TB Green Premix Ex Taq II (Takara Bio, Tokyo, Japan). *AtActin* was used as the internal control. Primer sequences used for qRT-PCR in this study are shown in Table S1.

## Supplementary Material

Web_Material_uhaf352

## Data Availability

The data that support the findings of this study are available in the supplementary material of this article. Genomic and single-cell sequencing data generated in this study have been deposited in the National Genomics Data Center under BioProject accession number CRA033556 and are publicly accessible.
