# Supplementary material for: Exploring the developmental mechanisms of tea plant trichomes using genomics and single-cell transcriptome sequencing
Source: Hortic Res. 2025 Dec 9;13(3):uhaf352. doi: 10.1093/hr/uhaf352 (PMC13034034; doi:10.1093/hr/uhaf352)
Supplement: Web_Material_uhaf352 [file web_material_uhaf352.docx]

**Exploring the Developmental Mechanisms of Tea Plant Trichomes Using Genomics and Single-Cell Transcriptome Sequencing**


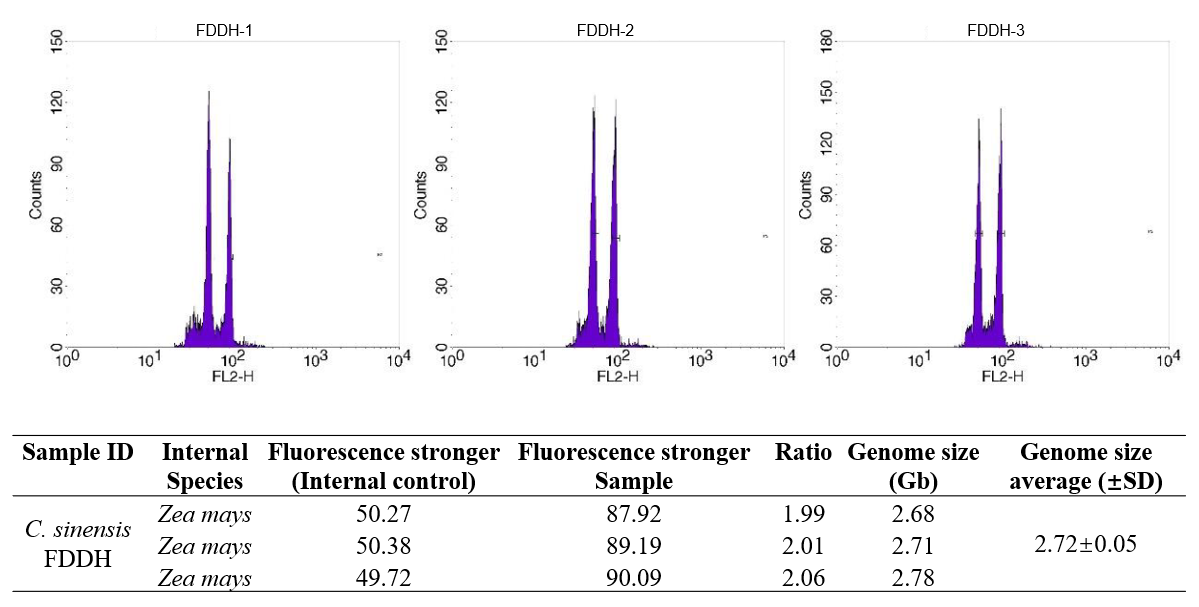


**Supplementary Figure 1.** The histogram of flow cytometry analyses of *C. sinensis* FDDH. Zea mays was used as an internal reference in Flow cytometry analyses, and 3 replications were set. The genome size of C. sinensis FDDH was estimated nearly 2.72Gb by comparing to the fluorescence stronger of internal control and the results of karyotype analysis (triploid). Internal control Zea mays B73 (reference Genome size 2.3G).


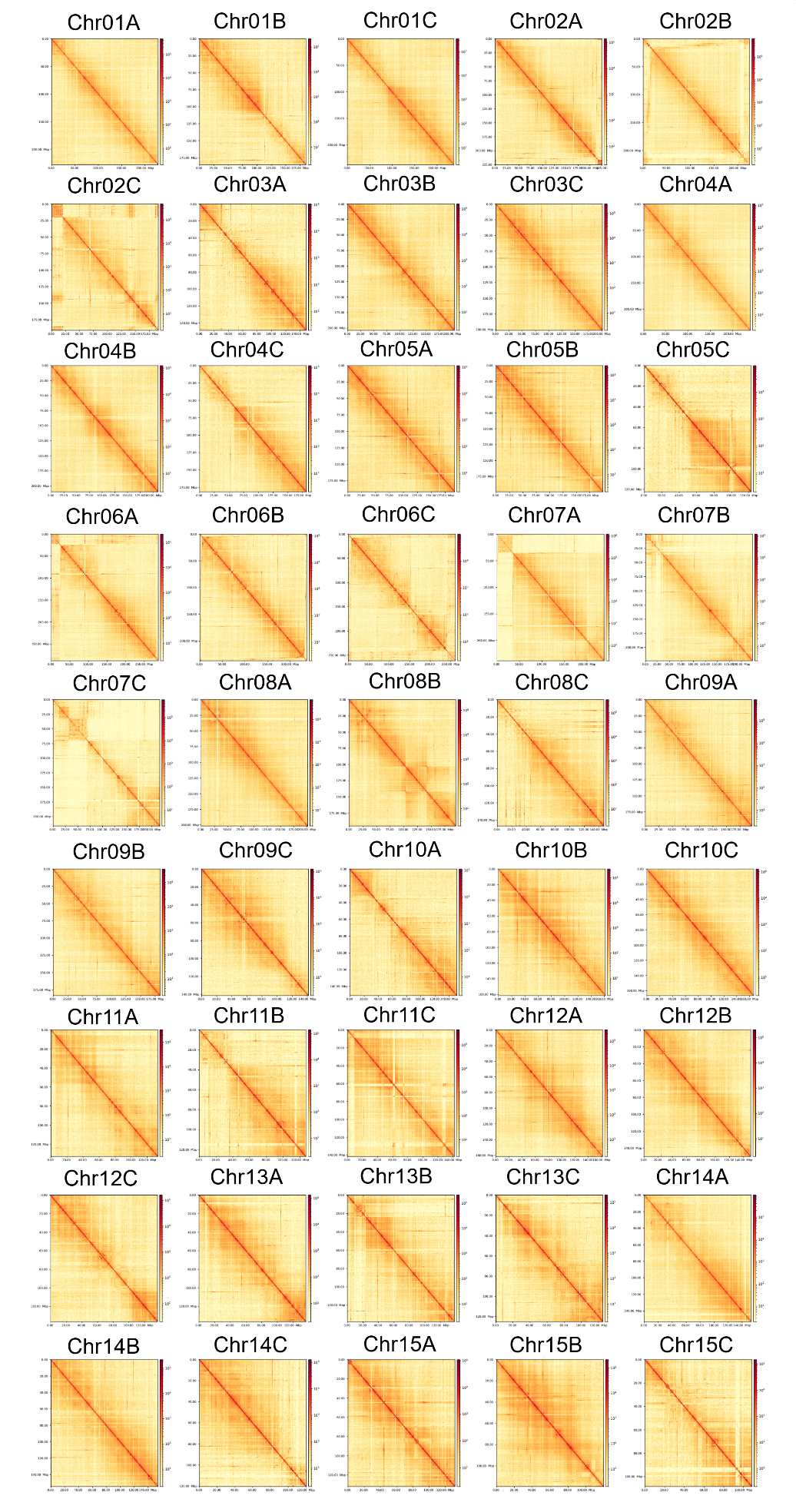


**Supplementary Figure 2.** The Hi-C interaction heatmap plot for each *C. sinensis* FDDH chromosoes.


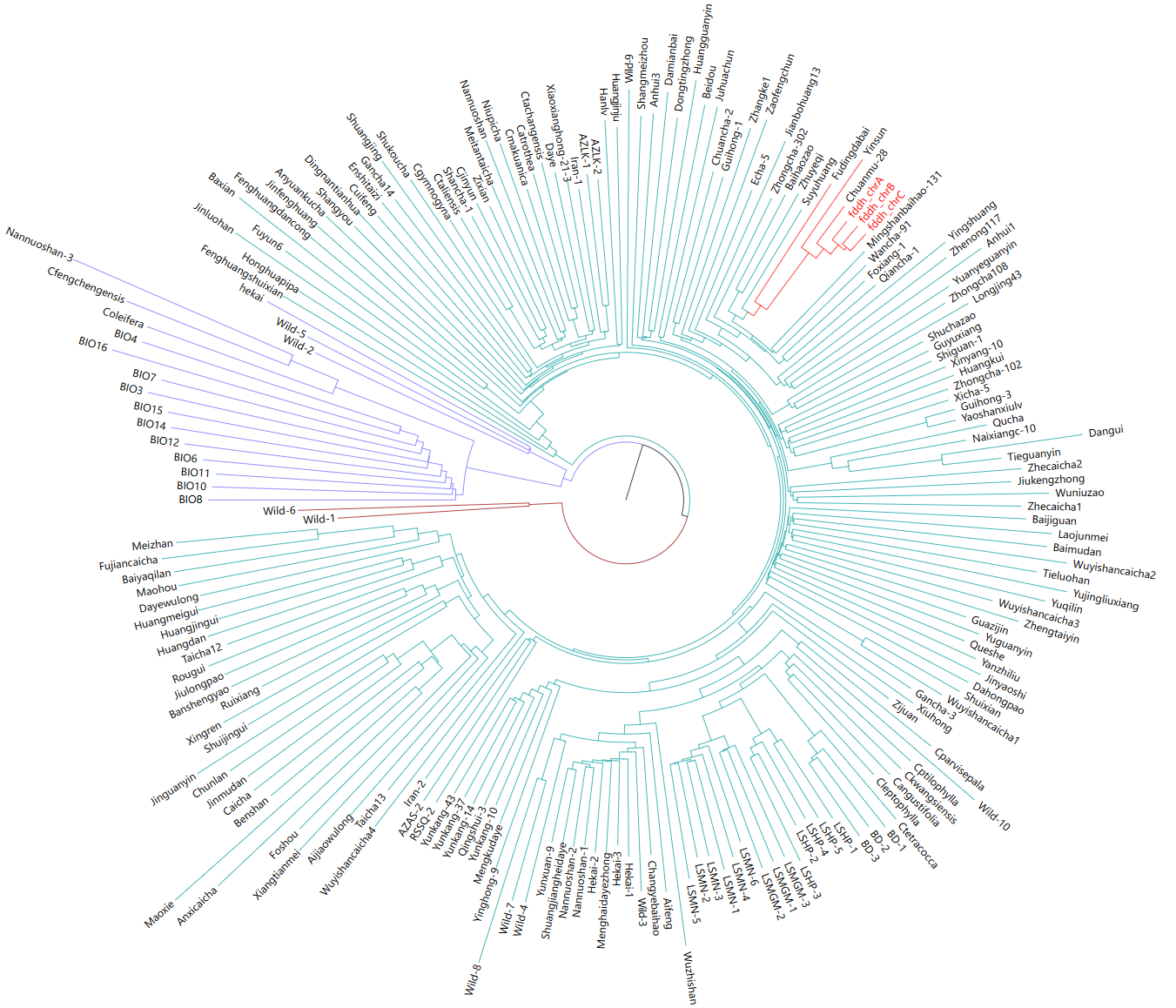


**Supplementary Figure 3.** The maximum-likelihood phylogenetic tree diagram, including three sets of chromosome complements of the *C. sinensis* FDDH and 196 varieties of tea trees.


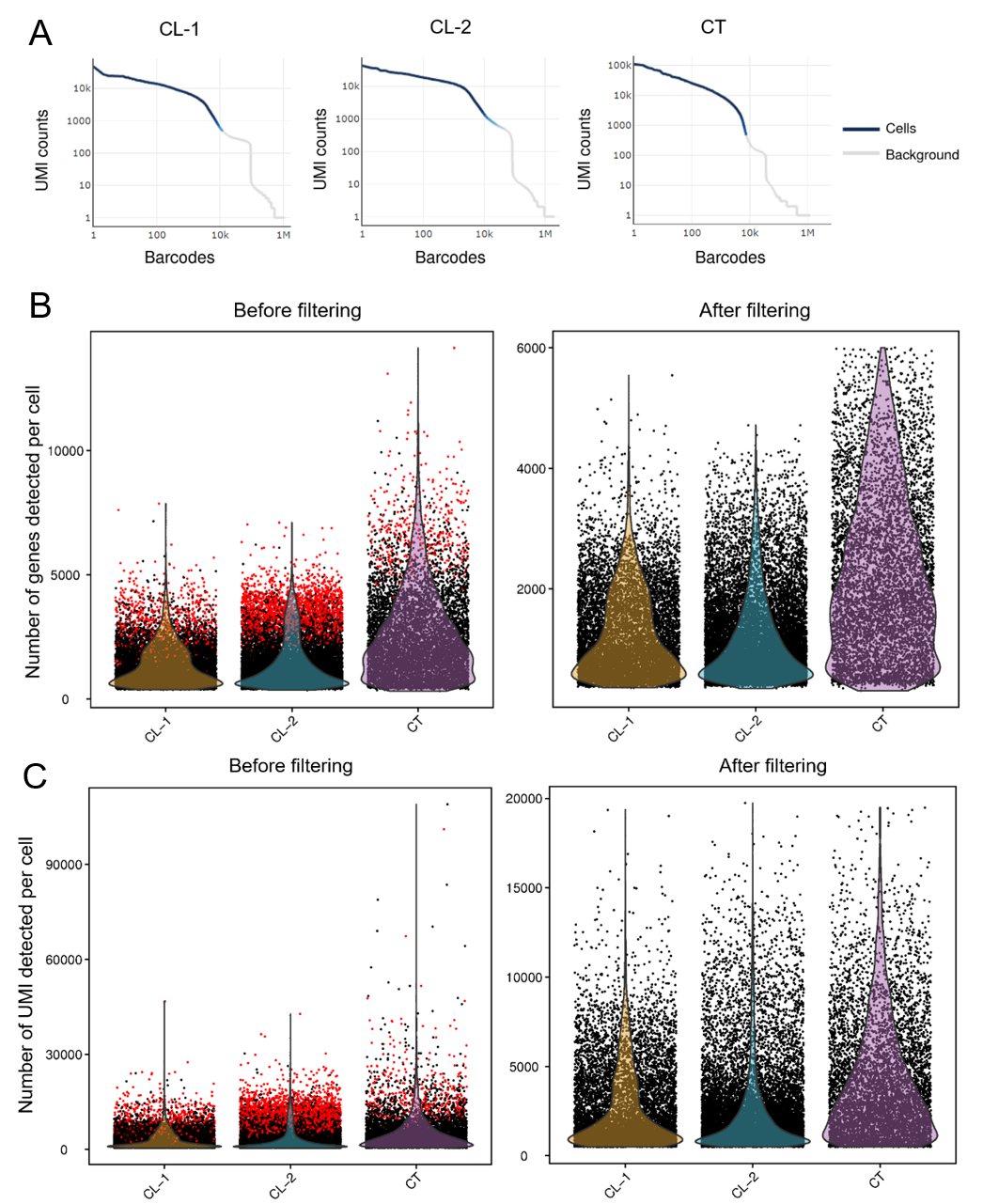


**Supplementary Figure 4.** The identification of effective cells in single-cell transcriptome atlas construction.

(A) Barcode rank-plot of effective cell identification; (B) Comparison of the number of cellular genes before and after filtration; (C) Comparison of the number of UMIs in cells before and after filtration. Red dots indicate multicellular GEMs and the black dots represent single cell GEMs.


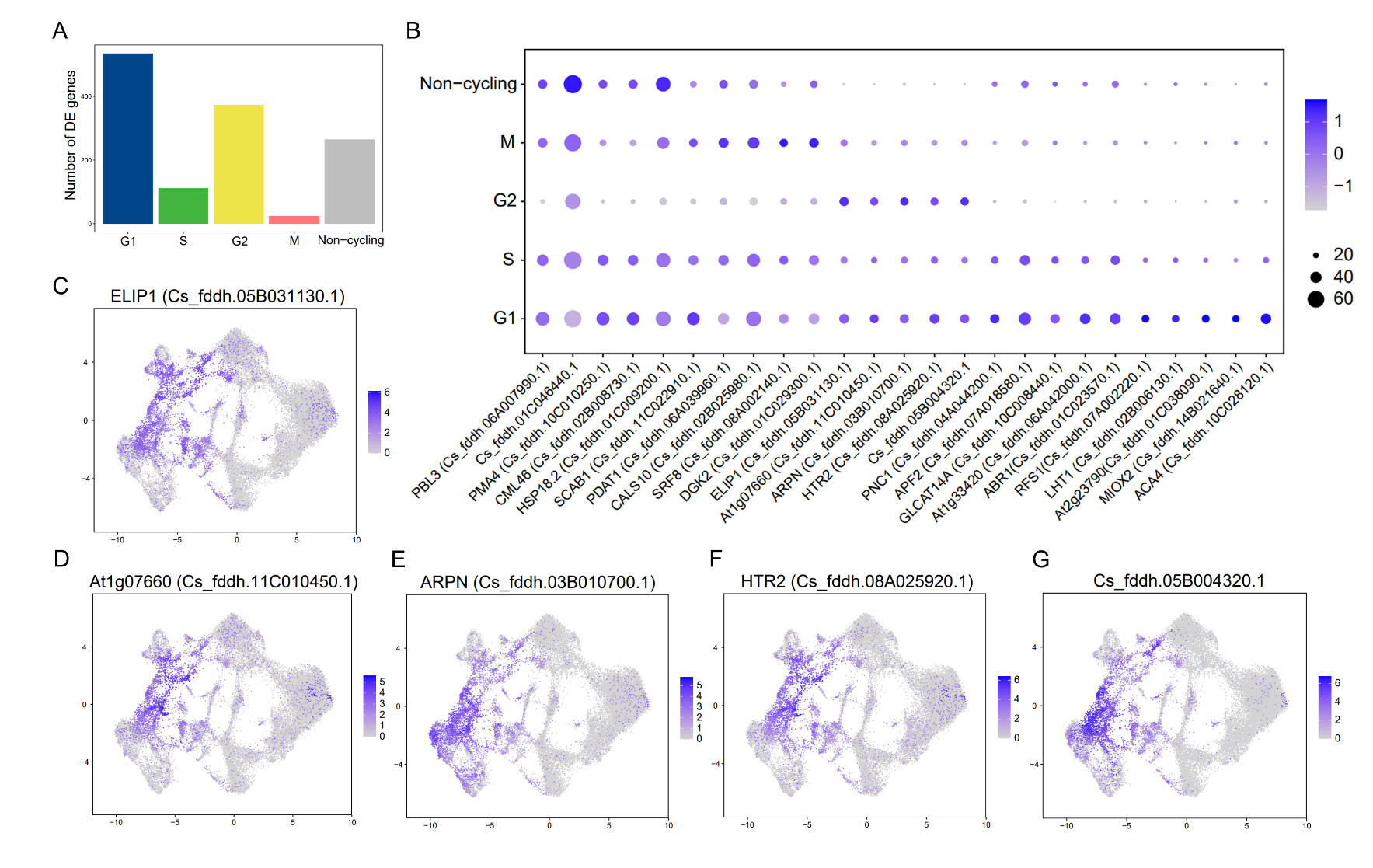


**Supplementary Figure 5.** The expression of cell cycle phase-specific marker genes.

(A) Statistical histogram showing the number of significantly upregulated genes in cells across different cell cycle stages; (B) Bubble plot depicting the expression distribution of marker genes across different cell cycle phases. Note: A larger circle in the legend indicates a larger proportion of the number of cells in that cluster expressing the gene, and vice versa; Legend values represent the expression abundance of a gene, with larger values indicating a higher abundance of gene expression in that cell and vice versa; (C-G) UMAP visualization of the distribution of key marker genes in the G2 cell cycle phase.


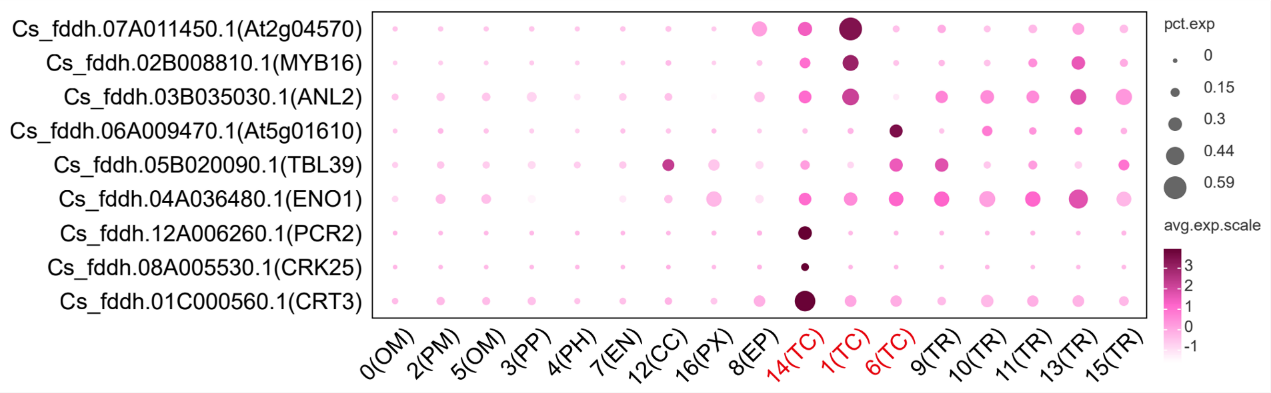


**Supplementary Figure 6.** Bubble plot showing expression distribution of marker genes in the TC clusters. Note: A larger circle in the legend indicates a larger proportion of the number of cells in that cluster expressing the gene, and vice versa; Legend values represent the expression abundance of a gene, with larger values indicating a higher abundance of gene expression in that cell and vice versa.


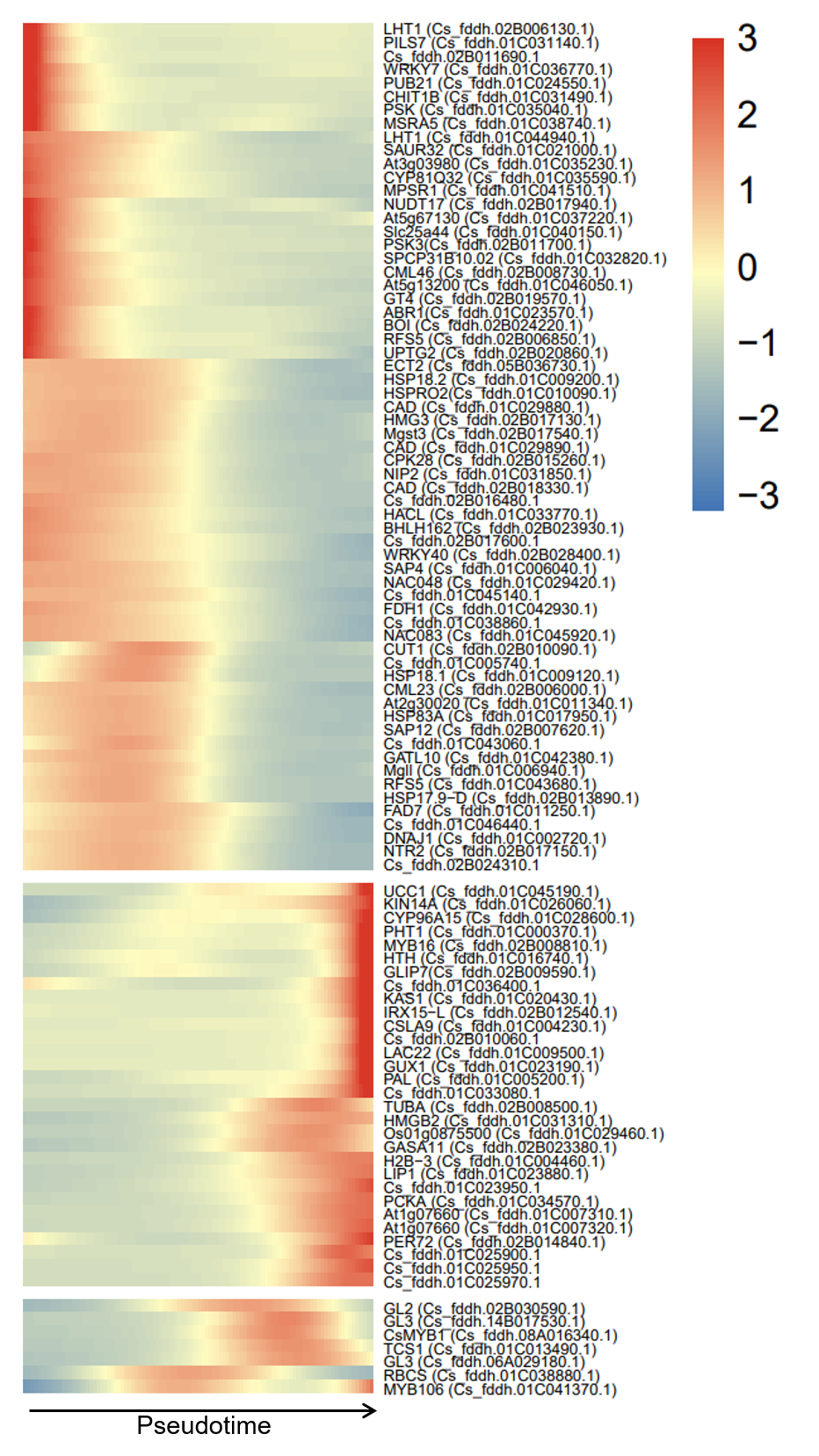


**Supplementary Figure 7.** Heatmap of the top 100 most significantly differentially expressed genes across the pseudotime axis. Note: Legend values represent the expression abundance of a gene, with larger values indicating a higher abundance of gene expression in that cell and vice versa.


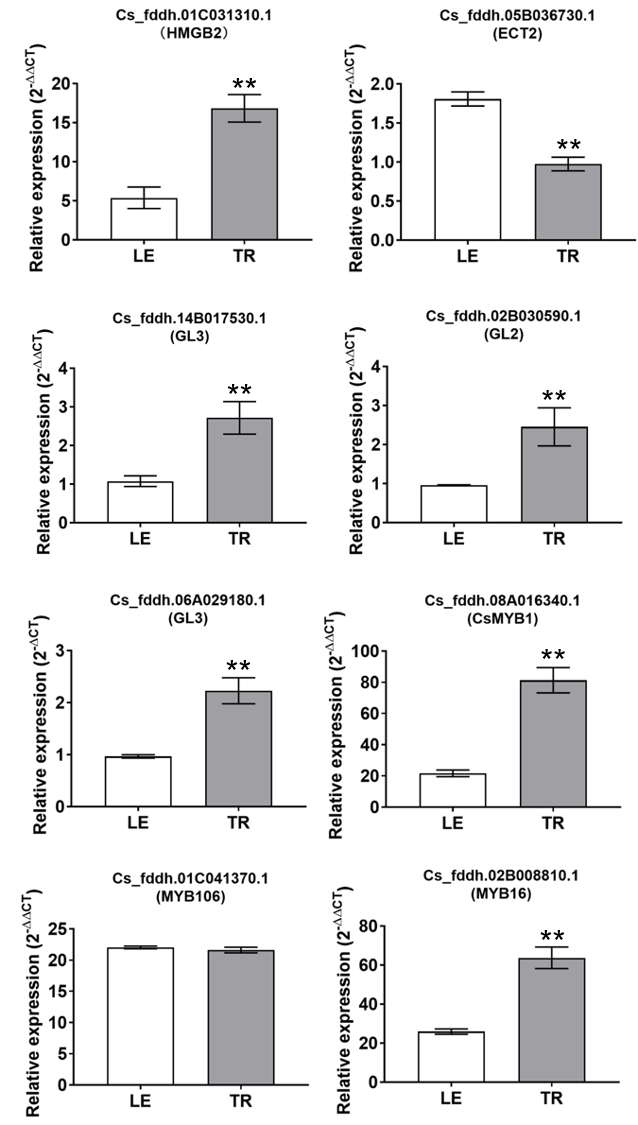


**Supplementary Figure 8.** RT-PCR results of genes regulating trichome development. Note: ** indicates statistically significant t-test relative to the lower epidermis of leaf (LE), *P* < 0.001.


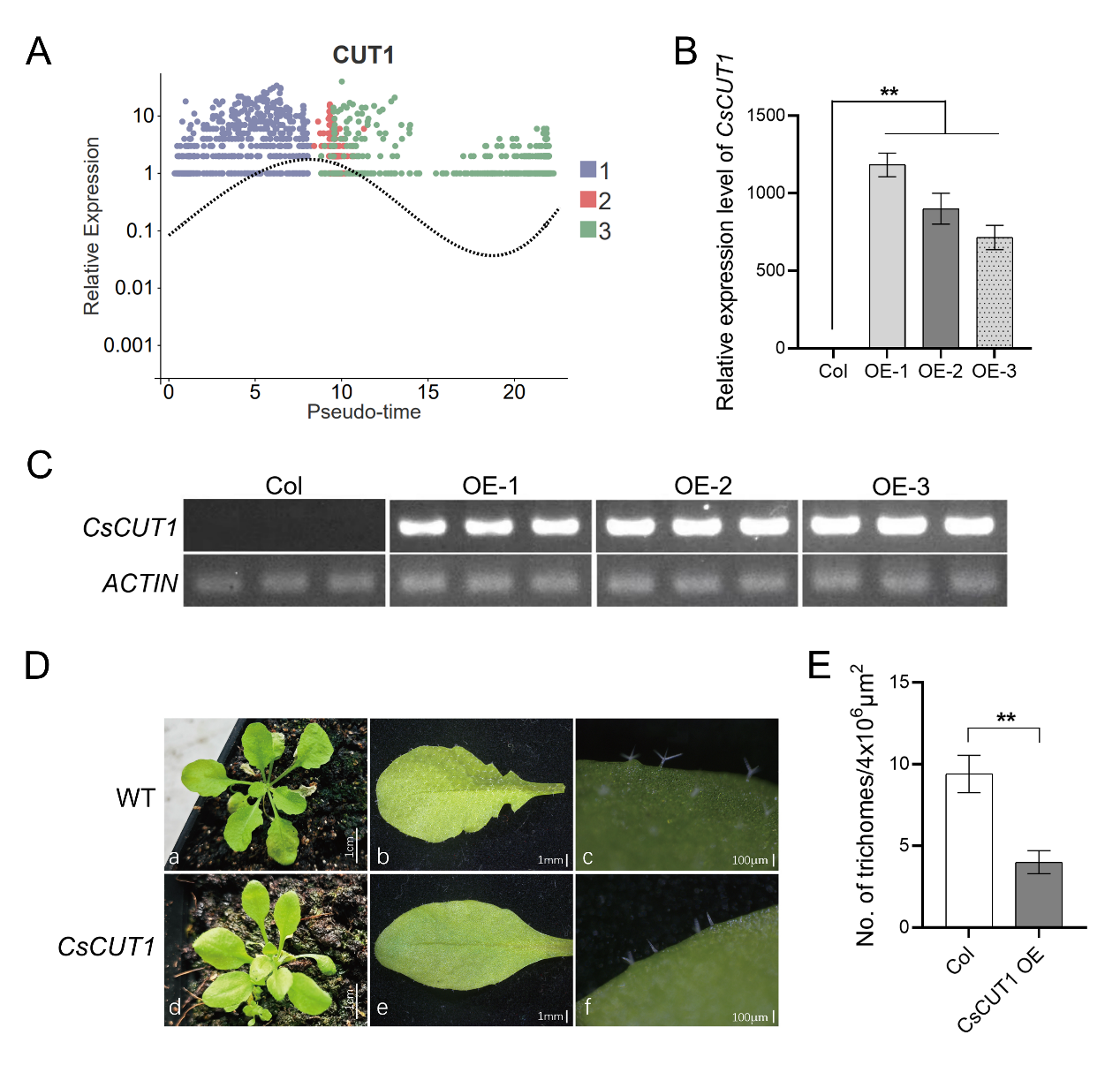


**Supplementary Figure 9.** Phenotypes of 35S:: *CsCUT1*-overexpression in Arabidopsis.

(A) Scatterplot of expression trends for *CUT1* across the pseudotime trajectory. Note: Different colors indicate the states of pseudotime; Each dot represents a cell; (B) Bar chart illustrating the relative expression levels of *CUT1* gene in Arabidopsis thaliana plants overexpressing 35S::*CUT1* compared to the wild-type plants; (C) Agarose gel image showing PCR bands representing *CUT1* gene expression in Arabidopsis thaliana plants overexpressing 35S::*CUT1* compared to the wild-type plants; (D) Phenotypic observation of leaves from *CsCUT1*-OE transgenic plants. Four-week-old whole Arabidopsis plants (a & d) and their first pair of rosette leaves (b-c & e-f). (E)The number of trichomes in Arabidopsis thaliana following *CsCUT1* overexpression. **: Indicates statistical significance by t-test compared with wild-type Arabidopsis thaliana, *P* < 0.001.


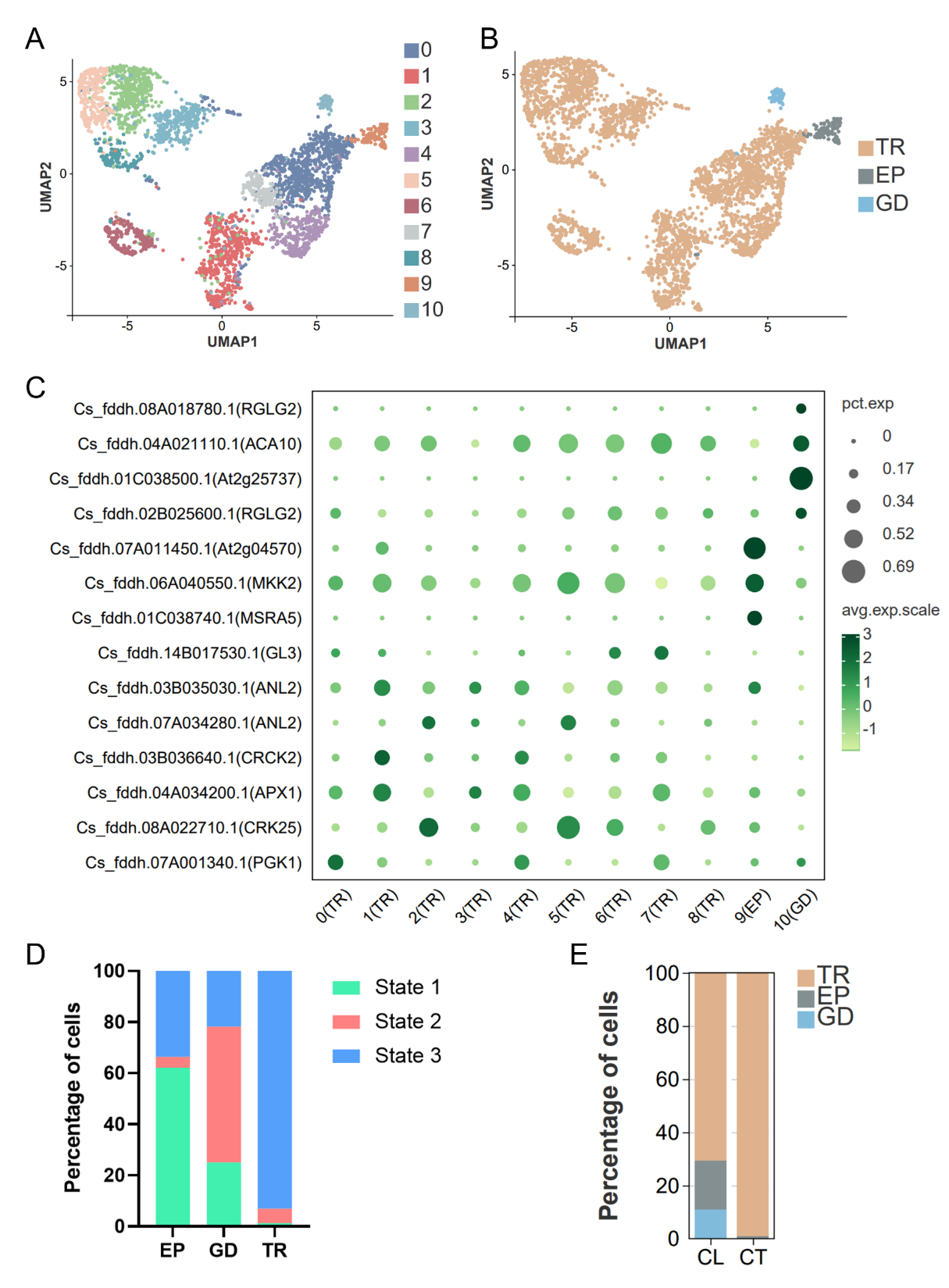


**Supplementary Figure 10.** Identification of tissue types comprising the rTR cluster.

(A) UMAP representation of the re-clustered cells within the rTR cluster; (B) UMAP depiction of the distribution of different tissue types within the rTR cluster; (C) Bubble plot illustrating the marker genes specific to different tissue types within the rTR cluster; A larger circle in the legend indicates a larger proportion of the number of cells in that cluster expressing the gene, and vice versa; Legend values represent the expression abundance of a gene, with larger values indicating a higher abundance of gene expression in that cell and vice versa; (D) Relative proportions of EP, GD, and TR cell types across various differentiation states; (E) Comparison of the distributions of EP, GD, and TR cell types in CL and CT samples.


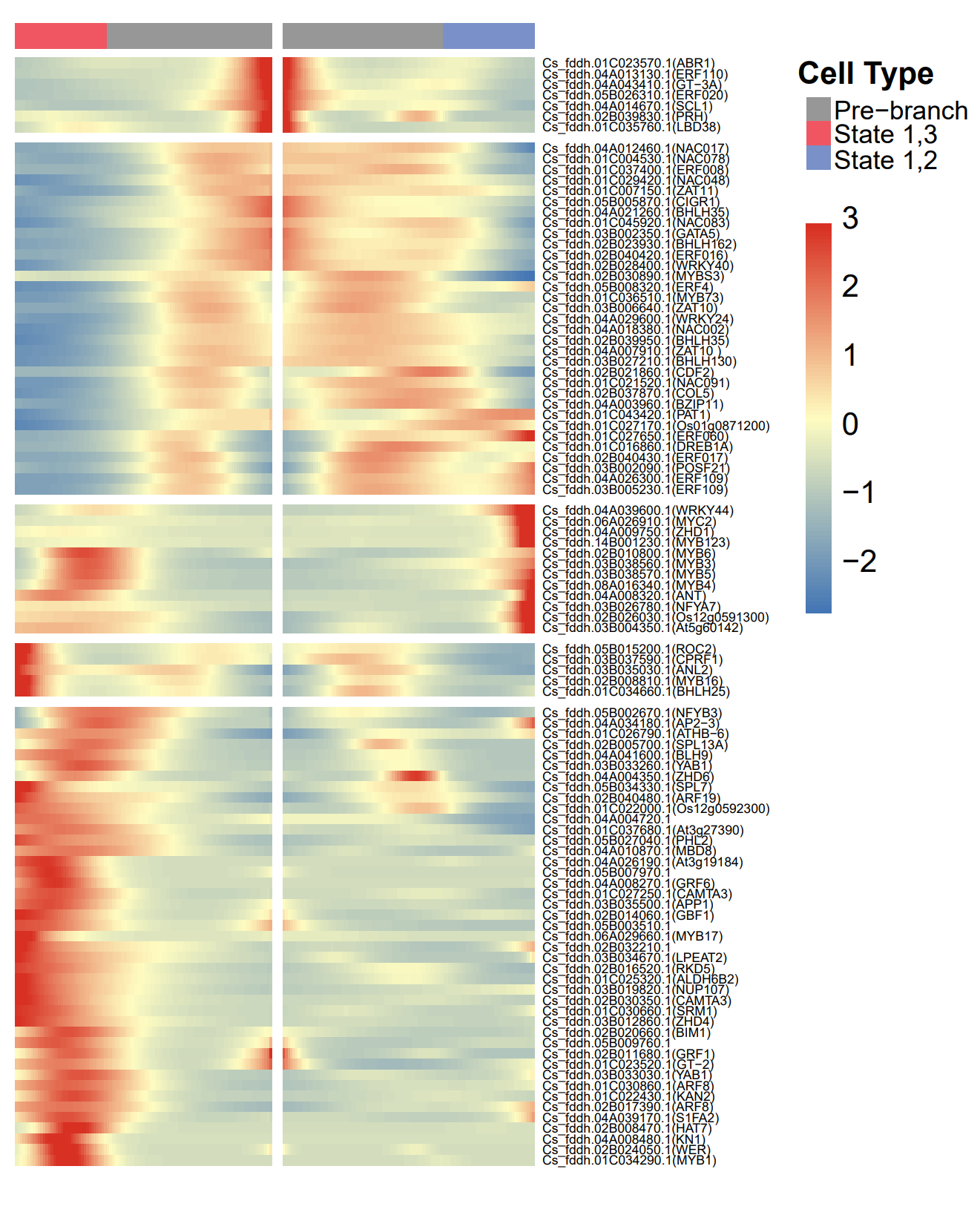


**Supplementary Figure 11.** Heatmap of expression levels for the 100 most significantly differentially expressed TFs associated with cell differentiation fates. Note: Legend values represent the expression abundance of a gene, with larger values indicating a higher abundance of gene expression in that cell and vice versa.


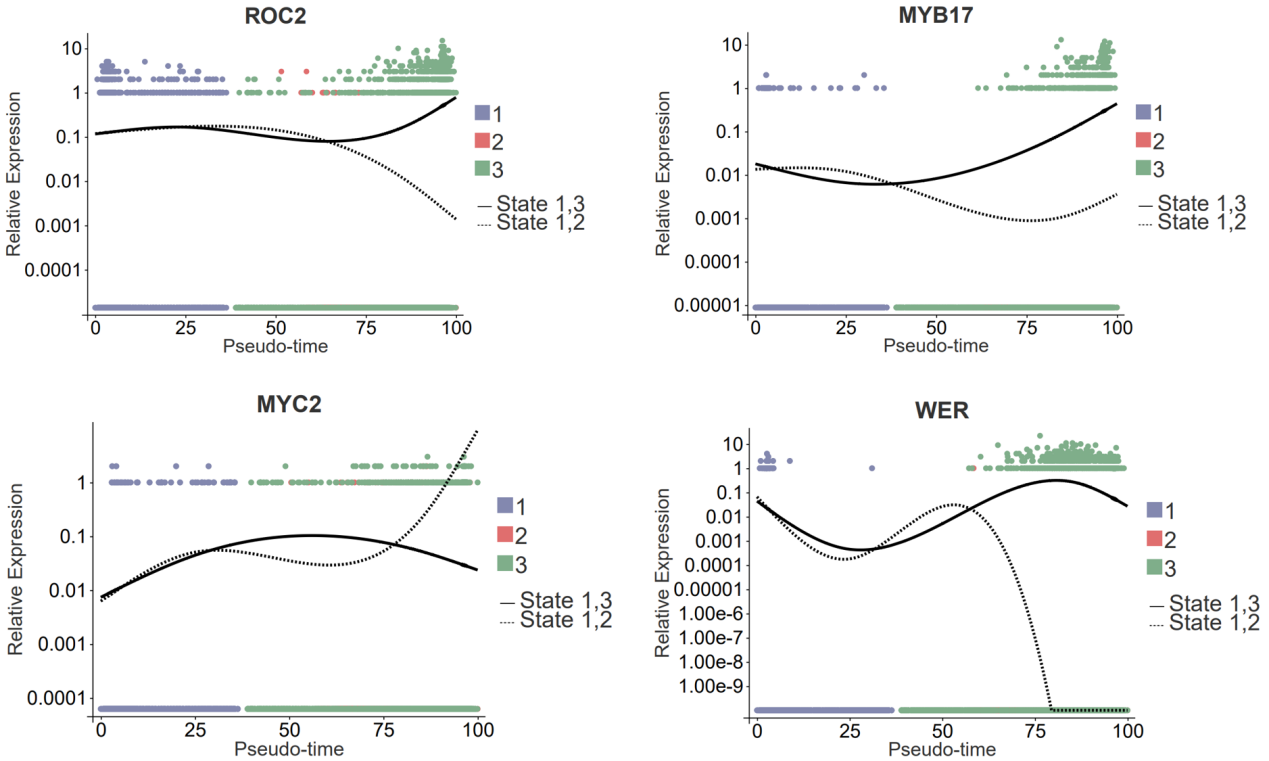


**Supplementary Figure 12.** Scatterplot of expression patterns for TFs associated with cell differentiation fates. Note: Different colors indicate the states of pseudotime; Each dot represents a cell.
